# Guava Leaf Extract Inhibits Quorum-Sensing and *Chromobacterium violaceum* Induced Lysis of Human Hepatoma Cells: Whole Transcriptome Analysis Reveals Differential Gene Expression

**DOI:** 10.1371/journal.pone.0107703

**Published:** 2014-09-17

**Authors:** Runu Ghosh, Bipransh Kumar Tiwary, Anoop Kumar, Ranadhir Chakraborty

**Affiliations:** OMICS Laboratory, Department of Biotechnology, University of North Bengal, Siliguri, West Bengal, India; Johns Hopkins University, Bloomberg School of Public Health, United States of America

## Abstract

Quorum sensing (QS) is a process mediated via small molecules termed autoinducers (AI) that allow bacteria to respond and adjust according to the cell population density by altering the expression of multitudinous genes. Since QS governs numerous bioprocesses in bacteria, including virulence, its inhibition promises to be an ideal target for the development of novel therapeutics. We found that the aqueous leaf extract of *Psidium guajava* (GLE) exhibited anti-QS properties as evidenced by inhibition of violacein production in *Chromobacterium violaceum* and swarming motility of *Pseudomonas aeruginosa*. The gram-negative bacterium, *C. violaceum* is a rare pathogen with high mortality rate. In this study, perhaps for the first time, we identified the target genes of GLE in *C. violaceum* MTCC 2656 by whole transcriptome analysis on Ion Torrent. Our data revealed that GLE significantly down-regulated 816 genes at least three fold, with *p* value≤0.01, which comprises 19% of the *C. violaceum* MTCC 2656 genome. These genes were distributed throughout the genome and were associated with virulence, motility and other cellular processes, many of which have been described as quorum regulated in *C. violaceum* and other gram negative bacteria. Interestingly, GLE did not affect the growth of the bacteria. However, consistent with the gene expression pattern, GLE treated *C. violaceum* cells were restrained from causing lysis of human hepatoma cell line, HepG2, indicating a positive relationship between the QS-regulated genes and pathogenicity. Overall, our study proposes GLE as a QS inhibitor (QSI) with the ability to attenuate virulence without affecting growth. To the best of our knowledge, this is the first report which provides with a plausible set of candidate genes regulated by the QS system in the neglected pathogen *C. violaceum*.

## Introduction

With the increase in the number of multi-drug-resistant pathogenic bacteria worldwide, there is a dire need for developing strategies to fight bacterial infections. The indiscriminate use of novel antibiotics that interfere with the metabolism of bacteria have only added to this number. Since Quorum Sensing (QS) regulates many virulence determinants of various pathogens, it has emerged as an attractive target to control their pathogenicity [1], [2], [3]. QS is a cell-to-cell communication mechanism, regulated by small diffusible signalling molecules termed autoinducers (AI), which allows bacteria to respond and adjust their needs in a population density-dependent manner by altering the expression of multitudinous genes [4], [5], [6]. The AIs used by Gram-negative bacteria are known as *N*-acyl homoserine lactones (AHLs), while Gram-positive bacteria utilize post-translationally modified oligopeptides as signaling molecules [7], [8]. In most Gram-negative bacteria, QS systems are based on LuxI/LuxR homologues. The LuxI homologues encode an AHL synthetase involved in the synthesis of signal molecules, and the LuxR homologues encode the transcription regulatory protein which, upon binding of the cognate signal molecules, activates the transcription of the QS target genes [9].

*Chromobacterium violaceum*, an opportunistic pathogen, is a free-living, gram-negative, facultative anaerobic β-proteobacterium commonly found in water and soil in the tropical and subtropical regions [10]. In human, *C. violaceum* infection is rare, but this may be attributed to under-reporting of such cases in areas where the risks of exposure are high and diagnostic facilities are scarce [11]. In spite of this, more than 150 cases of infection were reported in tropical and subtropical regions, including India, where *C. violaceum* is normally found [12]. This rare infection is associated with a high mortality rate, between 60% and 80%, if not diagnosed at an early stage or treated correctly [11]. A recent case of a man from South India with septicemic *C. violaceum* infection and septic arthritis, who had a fatal outcome, was reported [13]. A likely explanation for the high mortality rate could be the resistance of *C. violaceum* to a wide range of antibiotics and to other mech­anisms that pump out the cytotoxic drugs [14]. Thus, appropriate therapy is absolutely essential to control this neglected, potentially fatal infection. The strategy of controlling pathogens by interrupting its QS phenomenon was the prime focus of research in the recent years. The importance of quorum sensing in *C. violaceum* pathogenesis was demonstrated by the fact that QS-antagonist molecules protect the nematode *Caenorhabditis elegans* from *C. violaceum*-mediated killing [15]. The *C. violaceum* quorum-sensing system consists of the LuxI/LuxR homologues CviI/CviR, which controls virulence and the production of a variety of phenotypic characteristics that includes the production of the purple pigment, violacein, cyanide, chitinase and the antibiotic phenazine. The complete genomic sequence of *C. violaceum* ATCC 12472 has also revealed the presence of these QS-associated genes [16].

Inhibition of QS by some chemically synthesized compounds was identified but most of the QS inhibitors were isolated and characterized from plant sources [17], [18]. Crude extracts of many plant parts were shown to possess anti-QS activity using *C. violaceum* as a model bacterium [19], [20], [21]. Crude plant extracts are often found to be more effective than isolated constituents at an equivalent dose perhaps owing to positive interactions between components of whole plant extracts. This synergy may involve prevention of the active component from degradation by enzymes or facilitate transport across cell barriers that result in higher efficacy of the crude drug when compared with purified components [22], [23]. Hence, it is lately realized that crude extracts may possibly be the right strategy to treat multi-drug resistant pathogens as compared to the purified compound isolated from the same extract. In fact, the use of traditional herbal medicines is sometimes considered more effective than conventional drugs for the treatment of disease such as malaria [24]. It is proposed that the new generation of phytopharmaceuticals may enable successful use of herbal drug combinations to treat diseases in comparison to single active component [25].

*Psidium guajava* L. (Guava), widely distributed throughout India, belongs to the family Myrtaceae and is a well known traditional medicinal plant widely used in folk medicine [26], [27]. The leaf extracts of this plant were shown to possess anti-microbial [28], anti-inflammatory, antidiarrhoea [29], anti-oxidant [30], antimutagenic [31], anti- cancer [32], anti-diabetic [33] and anti-plaque [34] activities. However, no molecular mechanism of antimicrobial property of guava-leaf extract was explored.

To the best of our knowledge, this is the first attempt to reveal gene expression profile of *C. violaceum* with the aid of whole transcriptome analyses on Ion-Torrent in presence of guava-leaf extract (GLE). GLE inhibited QS-controlled genes and QS-regulated phenotypes without affecting the bacterial growth up to 24 h suggesting these effects to be unrelated to bacteriostatic or bactericidal effects. Furthermore, the down-regulation of the wide array of genes, including those encoding virulence factors, affect pathogenicity as revealed by the ability of GLE to arrest *C. violaceum* induced cell lysis of human hepatoma cells.

## Materials and Methods

### Bacterial strains

The *Chromobacterium violaceum* wild type strain MTCC 2656 and *Pseudomonas aeruginosa* MTCC 2297 were obtained from the Microbial Type Culture Collection Center (MTCC), IMTECH, Chandigarh, India. MTCC 2656 and MTCC 2297 cells were routinely cultured on Nutrient broth (NB, Hi-Media- M002) agar and Luria-Broth (LB, Hi-Media-M575) agar respectively and maintained at 37°C.

### Extraction of guava leaves

Leaves of *Psidium guajava* L. (Guava) were collected from the Centre of Floriculture and Agro Buisness Management (COFAM), University of North Bengal and extracted following standard method [35]. The leaves were washed thoroughly with sterile distilled water and rinsed with 70% (v/v) ethanol. Washed leaves were dried under sun initially and finally in the oven at 50°C for 1 hour. The dried leaves were crushed to fine powder, passed through an 80 mesh sieve and stored in a sealed plastic bag. To 50 gm of powder, 500 ml of sterile distilled water was added and the mixture was heated at 70°C for 1 hour and incubated at 30°C for 72 hours with shaking at regular intervals. The extract was filtered through a Whatman No. 1 filter paper (pore size 11 µm) and centrifuged at 10,000 rpm for 10 min. The resultant supernatant was freeze-dried using a lyophilizer (IIC Industrial Corporation, India). The dried sample was reconstituted with water, filter-sterilized (0.45 µm pore size, Sartorius Stedim, Germany) and tested for its ability to modulate quorum sensing in *Chromobacterium violaceum* strain MTCC 2656.

### Demonstration of Inhibitory effect of Guava leaf extract (GLE) on Quorum Sensing (QS) activity of *C. violaceum* MTCC 2656

The inhibition of QS-mediated violacein production in *C. violaceum,* by GLE, was studied by the agar well assay [36]. Sterile molten Nutrient agar (Hi-Media, India) was pour-plated with the cells of *C. violaceum* MTCC 2656. After solidification of the plates, wells were made in which 100 µl of the aqueous extract was placed, and incubated at 37°C for 24 h. In this assay system, inhibition of bacterial growth (if any) results in producing a clear circular zone around the well, while a positive result of quorum sensing inhibition is demonstrated by a colourless circular translucent zone, signifying growth of cells with no production of pigment, around the well, circumscribed by purple pigmented bacterial growth in the remaining part of the plate.

### Demonstration of anti-QS property of GLE using *Pseudomonas aeruginosa* MTCC 2297

To further confirm and or validate the anti-QS property of the GLE, motility assays of *Pseudomonas aeruginosa* MTCC 2297 were undertaken in petri dishes [37]. For this, LB media supplemented with 5% glucose and 0.5% agar containing either water (control) or 400 µg ml**^−^** ^1^ of GLE was poured in petri dishes. An inoculum of 10 µl of overnight grown MTCC 2297 was inoculated at the center of the plates. The inoculated plates were incubated at 37°C for 20****h and motility across the agar surface was visualized.

### Determination of QS-inhibitory concentration of GLE with reference to violacein production

Log phase cells of MTCC 2656 (2.5×10^6 ^CFU ml^−1^) were inoculated (1.0% inocula) into 10 ml volumes of sterile nutrient broth in flasks containing different concentrations of the GLE (100 µg ml^−1^to 1000 µg ml^−1^) and incubated at 37°C for 24 h with shaking. The control set was devoid of GLE. The violacein pigment formation in the flask was quantified following the method of Choo *et al*. [38]. Briefly, 1 ml culture from each flask was centrifuged at 13,000 rpm for 10 min. The culture supernatant was discarded and 1 ml of DMSO was added to the pellet. The solution was vortexed vigorously to completely solubilize violacein and centrifuged at 10,000 rpm for 10 min to remove the cells. The absorbance of the supernatant was read at a wavelength of 585 nm in a digital spectrophotometer (ThermoSpectronic UV 1). The maximum O.D._585_ value observed in case of GLE-untreated cells was considered as 100% production of violacein. The percentage (%) inhibition in violacein production was calculated as follows: % inhibition in violacein production = [(O.D._585_ value observed in the absence of GLE – O.D._585_ oberved in presence of a defined quantity of GLE)÷O.D._585_ value observed in absence of GLE]×100.

### Determination of growth and violacein production of *C. violaceum* MTCC 2656 in presence and absence of GLE

Growth of MTCC 2656 was quantified in terms of viable cell number present in the culture (dilution plating followed by counting CFUs) at different time interval [39]. Briefly, an overnight culture of MTCC 2656 (in NB medium), that had been inoculated with a freshly grown single colony, was diluted 100-fold into 10 ml NB medium and allowed to grow for 4 h to obtain log phase cells. The culture was then inoculated into 800 ml NB medium and divided into two portions, to one of which water (control) and to the other, GLE at a final concentration of 400 µg ml^−1^, was added. These were finally distributed in 10 ml aliquots per pre-sterilized 100 ml Erlenmeyer flask, and incubated at 37°C with shaking. At different time intervals, cultures were withdrawn from time-defined flasks (both control and test) for dilution plating onto NB agar plates as well as for the estimation of violacein by the method described above.

### Whole genome transcriptome analysis

The detail of the methodology is provided in File S1. In brief, RNA was isolated from control (cells grown without GLE) and experiment (cells grown with GLE) samples and cDNA Library was prepared using the Ion Total RNA-Seq Kit v2 (Catalog Number 4475936). Template preparation and enrichment was performed as per Ion OneTouch 200 Template Kit (Cat no. 4471263) and 200 base-read sequencing was performed using the Ion PGM 200 Sequencing Kit (Cat no 4474004) on ION TORRENT.

### Infection and morphological assessment of HepG2 cells by phase-contrast inverted microscope

HepG2 cells (Human hepatocellular liver carcinoma cell line), obtained from Cell Culture Collection, NCCS, Pune, were grown in 100 mm polyvinyl coated plates, in DMEM (Dulbeco’s Modified Eagle’s Medium) media with 10% FCS at 37°C in a humidified atmosphere containing 5% CO_2_. Cells (1×10^6^) were seeded in 60 mm plates in DMEM medium supplemented with 10 U/ml penicillinG and 100 µg ml^−1^ streptomycin. After 24 h, the media was removed and cells were washed thrice with phosphate buffer (PBS, pH 7.2) and grown in DMEM media without antibiotics until *C. violaceum* infection. Bacteria (MTCC 2656) were grown overnight at 37°C in NB either without or with 400 µg ml^−1^GLE. The following day, the bacterial cells were grown further in fresh media under the same conditions for 3 h. Finally, the untreated or GLE treated *C. violaceum* cells were diluted in DMEM with 10% FCS supplemented either with water or GLE, and added to HepG2 cells at the infectivity ratio of 10∶1. The plates were incubated at 37°C in an atmosphere of 5% CO_2_ and observed under inverted microscope (Olympus, ck40-slp) at 0 and 4 h of infection at 200X magnification and photographed. HepG2 cells incubated in DMEM containing GLE served as control.

### LDH cytotoxicity assay

Cytotoxicity induced in HepG2 cells was quantitated by measuring the release of the cytosolic enzyme lactate dehydrogenase (LDH) in the culture medium. For this, HepG2 cells were grown and exposed to *C. violaceum* as described above. After 4 h of incubation, supernatants were collected and evaluated for the presence of LDH using the LDH Cytotoxicity Assay Kit (Item No. 10008882, Cayman Chemical Company). The LDH activity (µU) was determined from the standard curve and the total LDH activity (µU ml^−1^) was calculated as value from LDH activity assay (µU)/sample volume assayed (ml).

### Statistical Analysis

All experiments were performed in triplicate. Results are expressed as mean value ± standard deviations (S.D) of three replicates and analyzed by using the software SPSS 15.0 for windows (SPSS Inc. Chicago, IL, USA). The statistical treatment of “Whole Transcriptome Analysis” data is elucidated in File S1.

## Results and Discussion

### Inhibition of violacein production in the presence of aqueous extracts of guava leaves

**Figure 1:**
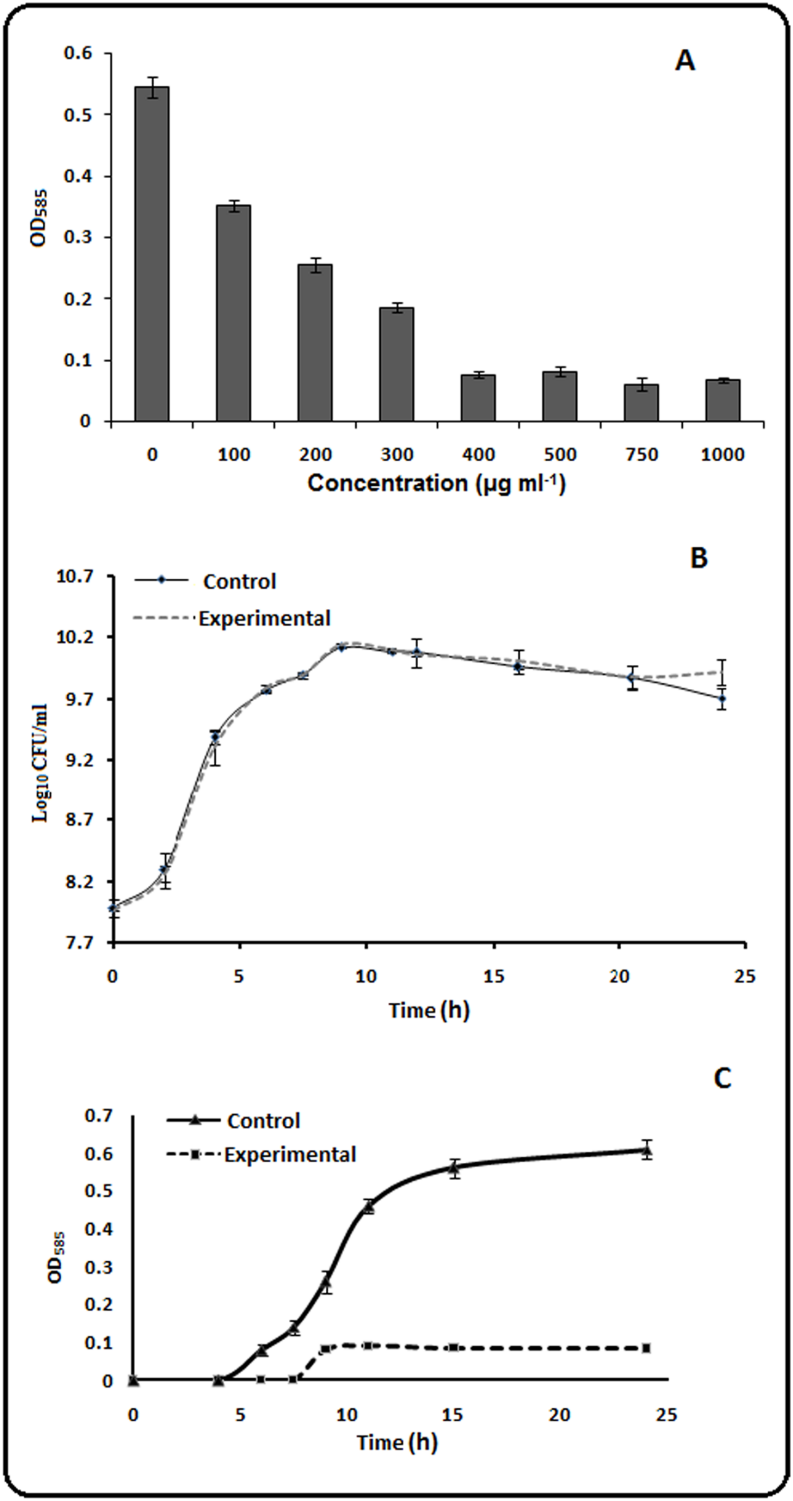
Effect of GLE on violacein production and growth of wild-type *C. violaceum* MTCC 2656 cells. **A.** Violacein production in the presence of different concentrations of GLE. **B.** Viable cell number in batch culture grown without (Control) or with (Experimental) supplementation of 400 µg ml^−1^GLE. **C**. Quantitation of violacein in batch culture grown without (Control) or with (Experimental) supplementation of 400 µg ml^−1^ GLE.

The best studied trait controlled by QS in *C. violaceum* is the production of the purple pigment, violacein [40]. To investigate QS inhibitory activity of aqueous extract of *Psidium guajava* L. leaves (GLE), its effect on violacein synthesis by *C. violaceum* MTCC 2656 was examined. A colourless transluscent zone around the zone of diffusion of GLE from the agar cup indicated growth of *C. violaceum* failing to produce violacein (purple pigment, Figure S1A). The production of violacein pigment at 24 h in the presence of varied concentration of GLE was quantified spectrophotometrically (Figure 1A). Violacein production was inhibited by 85% in presence of 400 µg ml^−1^ of GLE and the extent of inhibition remained similar at concentrations up to 1000 µg ml^−1^. Thus, the concentration of GLE to be used for further studies was selected as 400 µg ml^−1^. It has been documented in the Literature that nearly all parts of *Psidium guajava* L. tree, including fruits, leaves, bark, and roots, were used traditionally as a medicinal plant throughout the world for varied ailments [27]. The present study revealed that the guava-leaf extract was capable of inhibiting the quorum-dependent violacein production in *C. violaceum*.

### The growth of *C. violaceum* is unaffected in presence of GLE

The number of viable cell enumerated from growth studies in presence and absence of the GLE (400 µg/ml) showed no significant difference (Figure 1B). On the contrary, the violacein production was significantly inhibited under similar growth conditions (Figure 1C). It has been noted by earlier authors that the toxicity of putative quorum sensing inhibitors towards bacterial cells may be assessed by addressing three principal issues; that the QS inhibitory effects occur below the minimum inhibitory concentration (if the inhibitor exhibits antimicrobial property), the quorum-sensing inhibitory concentration used in the study does not affect the final cell density after a certain period of incubation, and the QS inhibitor does not grossly affect the kinetics of growth [41]. Our results show that the growth of *C. violaceum* remained unaffected but the quorum-dependent production of violacein was significantly reduced in the presence of GLE. These observations led us to conclude that the decrease in violacein production was not due to any form of growth inhibition of the cells in the culture medium at least up to 24 h of incubation. Similar results with respect to concentration-dependent decrease in violacein production without affecting bacterial growth were reported with extracts of various medicinal plants [42], [43]. Assuming that the quorum-inhibiting property of GLE should also be yielding in other bacteria that demonstrate quorum-dependent diverse phenotypes, we tested GLE for its activity using another test organism, *Pseudomonas aeruginosa*. The gram-negative bacterium *Pseudomonas aeruginosa* exhibits swarming motility which requires the bacteria to effectively work together via the process of quorum-sensing [37]. The cells of *P. aeruginosa,* MTCC 2297, in absence of GLE, formed tendrils migrating outwards from the point of bacterial inoculation, with continued branching as the bacteria moved farther from the center, while in presence of GLE, cells grew to form a localized colony in the center with no signs of swarming (Figure S1B). The ability of natural products to disrupt the quorum regulated swarming motility in *P. aeruginosa* was demonstrated earlier [44]. Thus, the manifestation of anti-QS property in the form of inhibition of swarming motility of *P. aeruginosa*, further provides evidence for the proposed quorum quenching property of GLE.

### Impact of GLE on the genome-wide gene expression of *C. violaceum*

The quorum-regulated gene expression has been extensively studied in gram negative pathogenic bacteria such as *P. aeruginosa* [45] and *Escherichia coli* [46]. However, *C. violaceum* transcriptomic studies have not yet attracted the similar attention. The increasing reports of *C. violaceum* cases [47] prompted us to unravel the status of gene expression in the presence or absence of GLE with a view to establish GLE as a QS inhibitory candidate for controlling the bacterial pathogenicity. Schuster *et al*. have observed that the timing affects the quorum-controlled gene expression in *P. aeruginosa* and most of the transcripts under QS-regulation were induced maximally at late log or stationary phase [48]. Thus, to get an insight into the effect of GLE on global gene expression of *C. violaceum*, we used 24 h grown cultures either in the absence (Control) or presence (Experimental) of 400 µg ml^−1^GLE.

**Figure 2:**
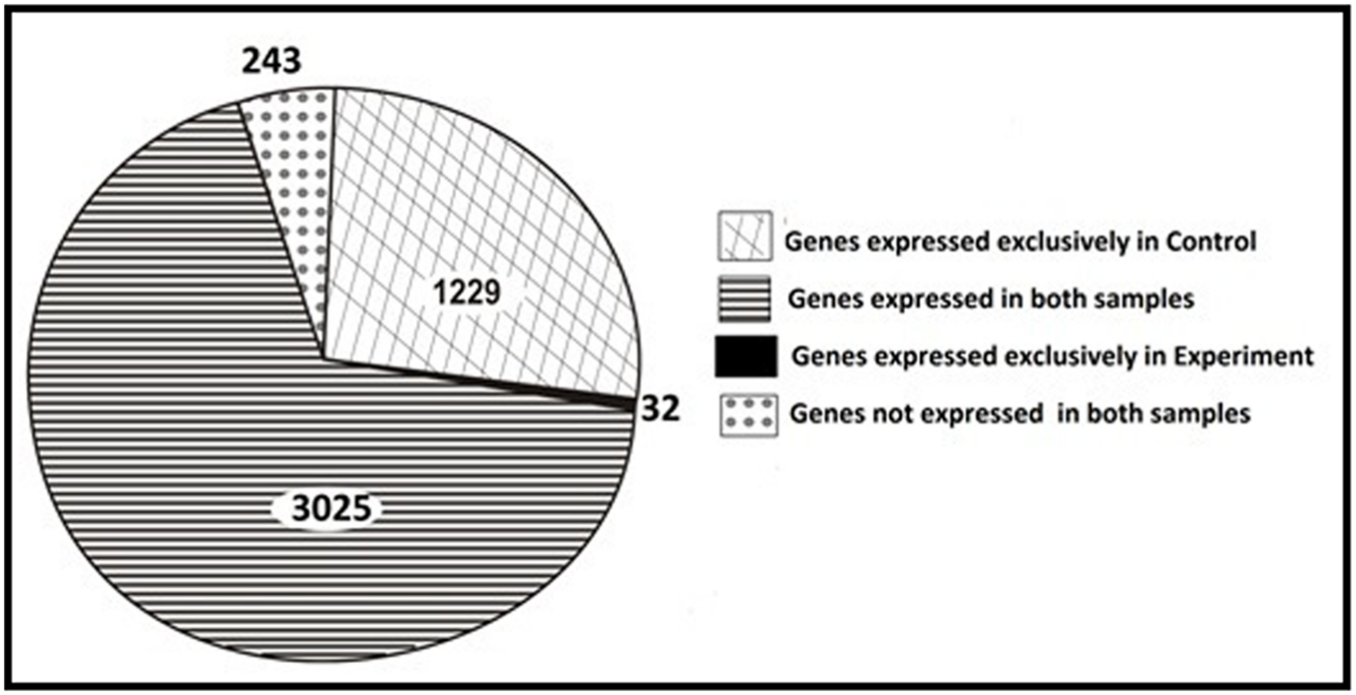
Profile of genome-wide gene expression in *C. violaceum* MTCC 2656 cells. Cells were grown in presence (experimental) and absence of 400 µg ml^−1^ GLE (control).

**Figure 3:**
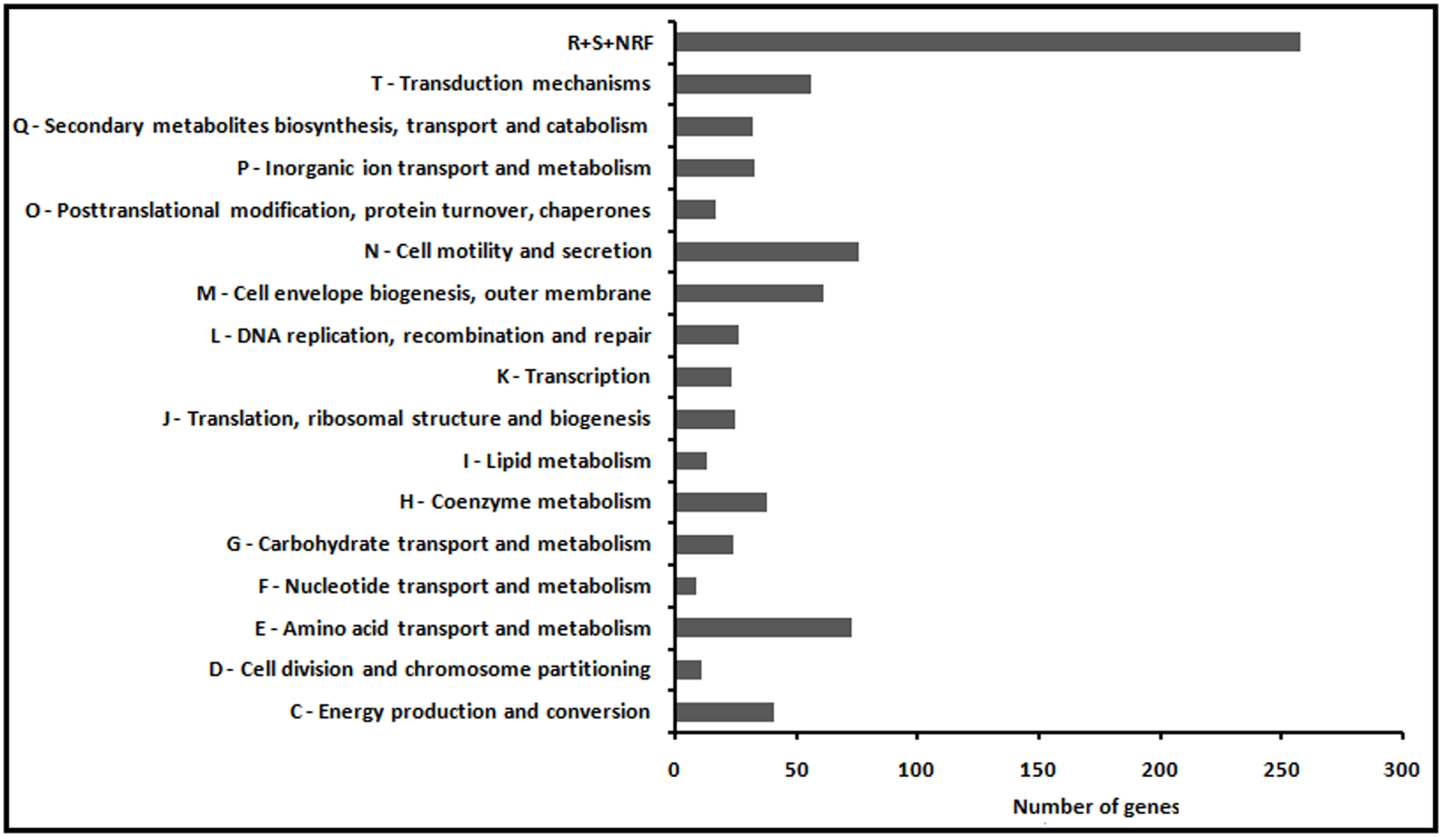
Differential expression of genes in *C. violaceum* in presence of GLE. The classification was based on Clusters of Orthologous Groups (COG) functional classification (R, General function prediction only; S, Function unknown; and NRF, No results found).

A total of 33,54,744 and 30,09,475 high quality Ion Torrent reads for Control and Experiment sample respectively were mapped on the reference genome of *Chromobacterium violaceum* ATCC 12472 with genome size of 4.75 Mb. There are 4,529 genes present in *C. violaceum* ATCC 12472 GFF file. The number of genes expressed in (i) both control and experiment; (ii) exclusively in control; (iii) exclusively in experiment; (iv) not expressed in both control and experiment were determined to be 3025, 1229, 32, and 243 respectively, as illustrated in Figure 2. Raw sequences were deposited at the NCBI Sequence Read Archive, under Bioproject accession number PRJNA243990 and SRA accession code SRP041018 (http://www.ncbi.nlm.nih.gov/bioproject/?term=PRJNA243990). The FPKM value for each gene was calculated for the combination of samples Control and Experiment. These FPKM values (FPKM_control and FPKM_experiment) were further used to calculate the log fold change as log_2_ (FPKM_experiment/FPKM_control). Moreover, uncorrected *p*-value of the test statistic for each gene was also computed. Using a stringent log_2_ threefold cut-off with *p*-value≤0.01, we observed that 816 genes, scattered throughout the *C. violaceum* MTCC 2656 genome, comprising about 19% of the total genes expressed, were significantly down regulated in the presence of GLE (Table S1). The 816 differentially expressed genes were categorised into 17 groups (Figure 3) based on the *C. violaceum* ATCC12472 database from the Brazilian Genome Virtual Institute of Genomic Research (BRGene) (http://www.brgene.lncc.br). The category representing the largest percentage, ∼32% of the total 816 genes, encoded for the group of proteins assigned unclassified and unknown functions. The categories representing>6.5% belonged to the functional classes of proteins namely cell motility and secretion (9.31%), cell envelope biogenesis, outer membrane (7.47%), transduction mechanisms (6.86%) and amino acid transport and metabolism (8.94%). Among these, the first three groups of proteins are related to virulence in many pathogens since these processes mediate interaction of bacteria with their immediate environment and genes encoding virulence factors. GLE down-regulated genes like lyases (*argH, aspA*), dehydrogenase and hydratase (*usg, sdaA1*) and transporters (*sdaC, argT*), that encode products involved in metabolism and transport of amino acids. In addition, GLE down-regulated, about 4%, the genes involved in energy production and conversion, secondary metabolites biosynthesis, inorganic ion transport and metabolism, and coenzyme metabolism. The possibility of interference of GLE with one or several global regulators in *C. violaceum* resulting in differential expression of multiple genes cannot be ruled out. However, since GLE targeted virulent genes which are often QS controlled [49] and QS activated genes involved in uptake, synthesis and degradation of amino acids [50]; inhibited QS controlled phenotype, violacein production, without hampering growth of *C. violaceum* upto 24 h, pointed to the possible anti-QS property of GLE. Repression of genes related to cellular processes with no effect on growth was revealed by microarray analysis of *P. aeruginosa* gene expression in the presence of QS inhibitor furanone C-30 [51]. Earlier microarray studies with *E. coli* and *P. aeruginosa* have revealed that varied cellular functions are largely affected by the QS system [45], [46]. Both the gram negative bacteria have more than one QS regulatory system. The cross-talk between different quorum signal pathways lead to either synergestic or antagonistic effects. It is thus tempting to assume that *C. violaceum* may also possess multiple QS systems and any interference would thus lead to a global differential expression of genes as observed with GLE. Previous transcriptomic studies have shown that as much as>10% of the total genes in the genome of *P. aeruginosa* are QS regulated [45], however, we observed a higher percentage (19%) of differential gene expression. This could be explained either due to the presence of multiple components of GLE or if we assume that these genes are QS-regulated, the higher number of genes revealed in this study may be due to the fact that earlier groups detected QS regulons using specific mutants but our studies involves revelation of QS regulons using a putative QS inhibitor on wild type cells. Thus, although the data obtained does not reflect a definitive analysis of gene regulation by quorum sensing, perhaps for the first time, our results provide with a set of probable candidate genes under QS regulation in *C. violaceum* for further investigation.

### Differential expression of genes predicted to be under QS-regulation in *C. violaceum*

Cell to cell signaling in *C. violaceum* is based on the CviI/CviR circuit. At high cell density, the cytoplasmic quorum-sensing receptor, CviR, binds to the AHL autoinducer. The CviR-AHL complex then binds to DNA and activates transcription of a number of genes. The fact that GLE has the ability to induce break in the CviI/CviR circuitry is reflected phenotypically by the abolition of violacein production by *C. violaceum* in the growth medium. The gene responsible for violacein production, *vioA*, was log_2_>threefold repressed. Since the level of expression of *vioA* gene (FPKM_control value) in absence of GLE was low (perhaps owing to the experimental conditions), the comparison with FPKM_experiment value (in presence of GLE) was concluded as statistically non-significant. Our data revealed that GLE down-regulated the expression of major virulence factors (Table 1) such as, *lasA* (CV_2571), *lasB* (CV_0057), *hcnABC* operon (CV_1682 to CV_1684) and chitinase (CV_4240). The quorum regulated proteases LasA and LasB are capable of inactivating a wide range of biological tissues and immunological agents to allow the bacteria to invade and colonize host tissues [52], [53]. The *hcnABC* operon responsible for biosynthesis of hydrogen cyanide (HCN), a secondary metabolite and a potent inhibitor of cytochrome oxidase and several other metalloenzymes, is known to be under the control of quorum-sensing mechanism in *C. violaceum* [54]. The enzyme, chitinase, is hypothesized to be involved in blocking the growth of fungi with chitinous exoskeleton, allowing a competitive advantage to *C. violaceum* during colonization [55]. In addition to these secreted factors, transcription of gene encoding acyl carrier protein (ACP) synthase, *fabF* (CV_3412) and other ACP-encoding genes like *fabZ* (CV_2207) and CV_3413, were significantly repressed by GLE. These gene products are proposed to be involved in synthesis of AHLs [56]. Similar down-regulation of these genes in *P. aeruginosa* was observed in the presence of the QS inhibitor, furanone C-30 [51]. Moreover, C-30 also targets *P. aeruginosa* virulence but it does not affect the expression of gene clusters encoding the components of QS system. On the contrary, GLE not only affected virulence but significantly repressed the expression of both *cviI* (CV_4091) and *cviR* (CV_4090) genes, the basic constituents of *C. violaceum* QS system, suggesting direct transcriptional interference of GLE. A putative cviR binding site was detected in the *cviI* promoter region and so its expression was expected to be down-regulated. In addition, a number of other *cviR* -regulated genes based on the presence of an ideal CviR binding site coupled with genome scanning were predicted [57]. GLE down-regulated a subset of these genes, which included CV_1328 (methyl-accepting chemotaxis protein), CV_2321 (recN, DNA repair protein) and CV_3062 (enoyl-CoA hydratase). The downregulation of QS-controlled genes with no effect on bacterial cell growth led us to hypothesize the anti-QS property of GLE to be responsible for the observed differential gene expression.

**Table 1:** Significantly downregulated *C. violaceum* genes associated with quorum-sensing and pathogenicity, in presence of GLE.

ORF no.	Genename	Description	Control^1^	Expt^2^	log_2_(foldchange)	p value
***Constituent of Quorum sensing circuit***						
**CV_4091**	*cviI*	N-acyl homoserine synthase;autoinducer synthase,quorum sensing controlled system	171.85	19.44	−3.14	5.39E-005
**CV_4090**	*cviR*	transcriptional activator,LuxR/UhpA family of regulators	194.51	14.75	−3.72	3.04E-006
***Genes predicted to have cviR binding sites***						
**CV_1311**		hypothetical	46.41	1.6	−4.86	0
**CV_1329**	*sbcB*	exonuclease I	15.82	0.66	−4.59	0
**CV_1328**		methyl-accepting chemotaxis protein	39.91	2.86	−3.8	3.86E-006
**CV_1408**	*sdaA2*	serine dehydratase	23.25	2.67	−3.12	0
**CV_1444**		hypothetical	14.81	0.94	−3.98	0.01
**CV_2091**		putative tetR-family transcriptional regulator	23.69	2.89	−3.03	0.01
**CV_2321**	*recN*	DNA repair protein	19.88	0.54	−5.19	0
**CV_2434**		hypothetical	56.1	1.93	−4.86	0
**CV_2656**		cytochrome P450 hydroxylase	96.94	8.63	−3.49	7.18E-006
**CV_2716**		hypothetical	52.45	5.47	−3.26	4.07E-005
**CV_3062**		enoyl-CoA hydratase	32.72	1.31	−4.64	0
**CV_3300**	*treB*	protein phosphohistidine-sugar phosphotransferase	8.82	0.55	−4	0.01
**CV_4091**	*cviI*	autoinducer synthase	171.85	19.44	−3.14	5.39E-005
**CV_4092**		aldehyde dehydrogenase	20.7	1.39	−3.9	0
**CV_4142**	*hoxX*	HoxX-like protein	7.99	0.53	−3.9	0.01
**CV_4240**		putative chitinase	42.51	2.82	−3.92	2.08E-006
**CV_4379**		hypothetical inside biotin synthesis operon	16.18	0.61	−4.74	1.65E-005
**CV_4382**	*comF*	competence protein F	26.97	1.71	−3.98	0.01
***Quorum Sensing controlled genes***						
***hcn operon***						
**CV_1682**	*hcnC*	hydrogen cyanide synthase HcnC	187.54	6.8	−4.78	7.43E-009
**CV_1683**	*hcnB*	hydrogen cyanide synthase HcnB	600.99	4.65	−7.01	0
**CV_1684**	*hcnA*	hydrogen cyanide synthase HcnA	600.99	4.65	−7.01	0
***Protease***						
**CV_2571**	*LasA*	LasA protease precursor	175.34	13.11	−3.74	1.13E-006
**CV_0057**	*LasB*	class 4 metalloprotease	211.37	20.93	−3.34	1.09E-006
***Cellulose biosynthesis***						
**CV_2675**	bscC	cellulose synthase, subunit C	9.3	0.22	−5.39	0
**CV_2676**	bscZ	endo-1,4-D-glucanase	9.3	0.22	−5.39	0
**CV_2677**	bcsB	cellulose synthase, subunit B	9.3	0.22	−5.39	0
***Other genes related to virulence and pathogenicity***						
**CV_3824**		conserved hypothetical protein	64.28	5.74	−3.49	6.81E-005
**CV_3828**	*pilB*	type 4 fimbrial biogenesis protein	63.95	5.32	−3.59	5.99E-006
**CV_0829**	*pilQ*	type 4 fimbrial biogenesis protein PilQ	96.39	2.11	−5.51	1.01E-013
**CV_0830**	*pilP*	type 4 fimbrial biogenesis protein PilP	96.39	2.11	−5.51	1.01E-013
**CV_0832**	*pilN*	type 4 fimbrial biogenesis protein PilN	85.03	6.41	−3.73	0
**CV_0833**	*pilM*	type 4 fimbrial biogenesis protein PilM	130.37	15.8	−3.04	5.79E-005
**CV_0179**	*pilT*	twitching motility protein PilT	53.97	6.7	−3.01	0
**CV_0180**	*pilU2*	twitching mobility protein transport	32.64	2.57	−3.67	0
**CV_1458**	*pilU1*	twitching motility protein	41.43	5.17	−3	0
**CV_3112**	*pilV*	type-4 fimbrial biogenesis PilV transmembrane protein	79.2	2.24	−5.14	0
**CV_2618**	*sipC*	cell invasion protein	17.62	0.92	−4.25	0.01
**CV_2619**	*sipB*	cell invasion protein	116.65	3.16	−5.21	6.23E-005
**CV_2620**	*spaT*	surface presentation of antigens; secretory proteins	116.65	3.16	−5.21	6.23E-005
**CV_2198**	*pyrH*	uridylate kinase, Pyrimidine metabolism	40.96	4.63	−3.15	0
**CV_2205**	*ompH*	hypothetical protein, outer membrane protein	46.62	5.62	−3.05	0.01
**CV_2206**	*lpxD*	Lipopolysaccharide biosynthesis	41.56	4.12	−3.33	0.01
**CV_2207**	*fabZ*	3-hydroxyacyl-[acyl-carrier-protein]dehydratase, Fatty acid biosynthesis	41.56	4.12	−3.33	0.01
**CV_2209**	*lpxB*	lipid-A-disaccharide synthase, Lipopolysaccharide biosynthesis	29.44	3.69	−3	0
**CV_2210**	*rnhB*	ribonuclease HII	29.44	3.69	−3	0
**CV_2212**		hypothetical protein	561.06	41.05	−3.77	0
**CV_4338**	*ftsZ*	cell division protein	61.92	7.3	−3.08	9.58E-005
**CV_4340**	*ftsQ*	cell division transmembrane protein	41.07	1.13	−5.19	0
**CV_4341**	*ddlB*	D-alanine-D-alanine ligase, Peptidoglycan biosynthesis	41.07	1.13	−5.19	0
**CV_4344**	*ftsW*	cell division protein	25.93	3.05	−3.09	0
**CV_4345**	*murD*	Peptidoglycan biosynthesis	25.93	3.05	−3.09	0
**CV_4346**	*mraY*	Peptidoglycan biosynthesis	19.51	1.81	−3.43	0
**CV_4349**	*ftsI*	cell division protein FtsI	157.11	0.52	−8.25	3.40E-008
**CV_4350**		cell division protein	157.11	0.52	−8.25	3.40E-008
**CV_4351**	*mraW*	16 S rRNA (cytosine1402-N4)-methyltransferase	157.11	0.52	−8.25	3.40E-008
**CV_4352**		conserved hypothetical protein	157.11	0.52	−8.25	3.40E-008
**CV_0516**		Ca binding hemolysin	7.43	0.71	−3.39	3.36E-005
**CV_0360**		thermolabile hemolysin	25.86	2.28	−3.5	0
**CV_1989**		porin	257.04	24.41	−3.4	6.76E-006
**CV_3104**		porin	1207.93	95.97	−3.65	7.68E-006
**CV_3829**		porin	813.08	69.81	−3.54	6.04E-006
**CV_3342**		hemolysin III	274	26.88	−3.35	1.39E-005

1 gene expression in cells grown for 24 h.

2 gene expression in cells grown for 24 h in presence of 400 µg ml^−1^ of GLE.

### Status of expression of genes plausibly networked with quorum sensing mechanism

The pathogenicity of *C. violaceum* is still poorly understood. Cause and effect relationship between QS system and pathogenicity of *C. violaceum* is yet an enigma. On the basis of whole genome sequence of *C. violaceum*, a catalogue of genes encoding probable virulence factors was prepared [58]. These included the genes encoding *pil* proteins and genes involved in lipopolysaccharide (LPS) and peptidoglycan biosynthesis. The type IV pilus gene cluster encodes Type IV pili which are appendages emanating from the surfaces of several gram-negative bacteria that are associated with pathogenicity [59], [60]. The expression of a majority of the *pil* genes found in *C. violaceum*, namely *pil B*, *T*, *U2*, *U1*, *V* and *pilQPNM*, were inhibited by GLE. Transcription of *lpxB* (CV_2209) and *lpxD* (CV_ 2206) genes, responsible for LPS biosynthesis and *murA* (CV_0440) and *murD* (CV_4345), genes required for peptidoglycan biosynthesis, were significantly down-regulated in the presence of GLE. As found in other gram negative bacteria, the LPS and peptidoglycan of *C. violaceum* are associated with activation of immune response in the host, which induces secretion of inflammatory cytokines resulting in septic shock [58]. Very recently, genes encoding potential secreted virulence factors of *C. violaceum* were identified [61]. Among these, GLE targeted the hemolysins (CV_0516, CV_0360 and CV_3342) and porins (CV_1989, CV_3104, and CV_3829). Increased expression of hemolysin and porins in presence of quorum signal autoinducer 2 (AI-2), mediator of second signaling pathway, has previously been documented in *E.coli* [46]. While unravelling the virulence determinant for *C. violaceum* induced cell lysis, Miki *et al*. observed that the formation of pore structures on the host cell membrane results in cell death in hepatocytes [62]. The pore formation involved CipB (*sipB*, CV_2619), a translocator of the *Chromobacterium* pathogenicity islands 1 and 1a (Cpi-1/-1a). GLE significantly down-regulated the expression of the *sipB* gene and two other linked genes, *spaT* (CV_2620) and *sipC* (CV_2618), cell invasion protein. Transcription of a vast number of genes involved in flagellar biosynthesis (*flhB1*, *flhF*, *fliL*, *fliE*, *fliM*, *fliN*, *fliC2*, *fliC3*, *flgK1*, *flaG*, *flil1*, *fleN* and *fliA1*, RNA polymerase sigma factor for flagellar operon) and chemotaxis (*cheV1*, *cheR3*, *cheR1*, *cheA*, *cheA2*, *cheZ*, *trg*, *nahY*, *motA1*, *motB1* and *motB2*) were repressed by GLE. Regulation of flagellar genes by QS has been previously reported in other bacteria such as *Yersinia pseudotuberculosis* and *Vibrio harvei* [63], [64]. A decreased expression of *motA* was observed in QS-mutant strains of *E. coli* [49]. The results indicate that down-regulation of the expression of the varied genes, responsible for virulence and cell motility, by GLE, may alter *C. violaceum* pathogenicity.

*C. violaceum* genome consists of four genes involved in cellulose production: *bcsA*, *bcsB*, *bcsZ* and *bcsC* [65]. Interestingly, in the presence of GLE, expression of *bsc CZ* and *bcsB* (CV_2675-CV_2677) were down-regulated indicative of a low level of cellulose production. Bacterial biofilms consist of bacteria colonies embedded in their own extracellular matrix composed of exopolysaccharides (EPS). Cellulose has been identified as an exopolysaccharide (EPS) in the extracellular matrix produced by several bacterial species. Decreased cellulose production in the presence of GLE will thus lead to inhibition in the formation of biofilms. This emphasises the proposed QS inhibitory property of GLE in light of the finding that QS has been implicated as an important step in biofilm formation [5], [66].

Earlier studies on anti-QS activities of crude plant extracts in bacteria have focussed mainly on demonstrating inhibition of expression of well-established specific QS-induced gene(s) [67]. In *C. violaceum*, quantitative real-time -PCR assay for expression of specific genes, namely, AI synthetase gene (*cviI*) and one of the violacein biosynthetic genes (*vioB*), was performed in order to establish that sub-lethal concentration of antibiotics can inhibit QS in *C. violaceum* [68]. Validation of the claims of remedies resulting from consumption of crude plant extracts (e.g crude extracts of *Ginkgo biloba*) by transcriptome profiling of the affected organs (e.g liver of rats on a high-fat diet) have provided with the molecular basis of herbal therapy [69]. The outcome of the present transcriptomic data improvises quorum inhibitory property of crude extract of guava leaves, GLE, and provides with a list of probable quorum regulated genes in *C. violaceum* giving insights into the features of this versatile bacterium. The attenuation of virulence by GLE, as revealed by the differential gene expression data, compelled us to test the phenotypic manifestation of *C. violaceum* induced cytolysis of human hepatoma cell line in presence of GLE.

### GLE inhibits *C. violaecum*-induced cytotoxicity in HepG2 cells

**Figure 4:**
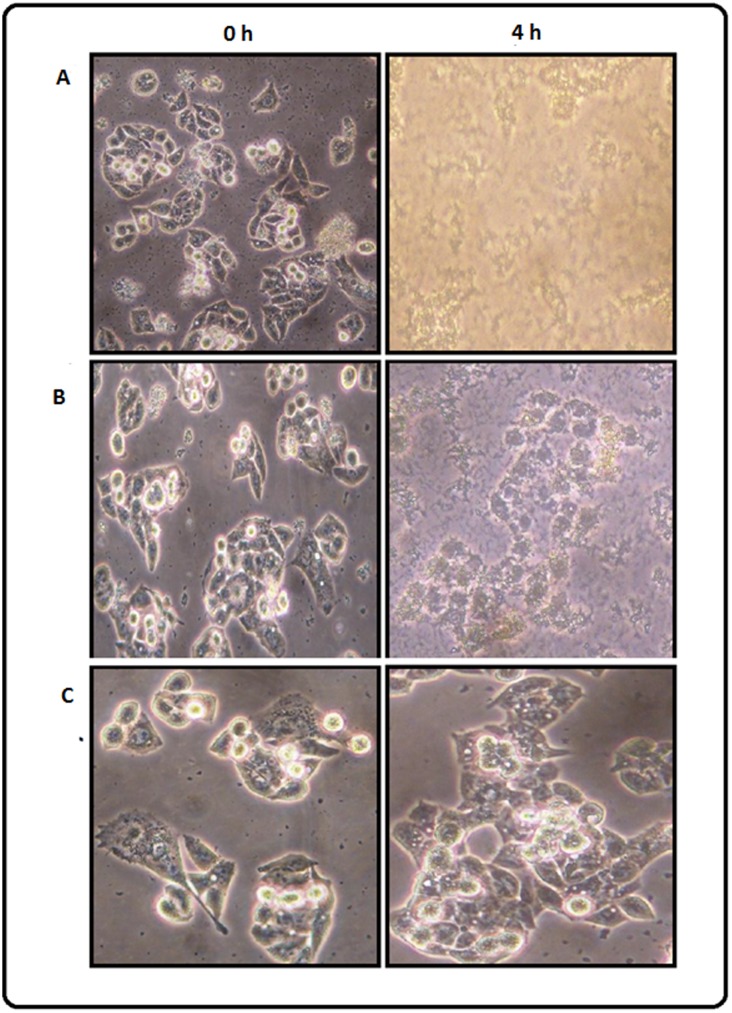
GLE inhibits *C. violaceum* induced cell-lysis of HepG2 cells. Phase contrast micrographs at 200X magnification showing (**A**) lysis of HepG2 cells after 4 h of infection with *C. violaceum* MTCC 2656 and (**B**) inhibition of lysis in presence of 400 µg ml^−1^ GLE. (**C**) growth of HepG2 cells in presence of GLE alone (to nullify any effect of GLE on HepG2 growth).

**Figure 5:**
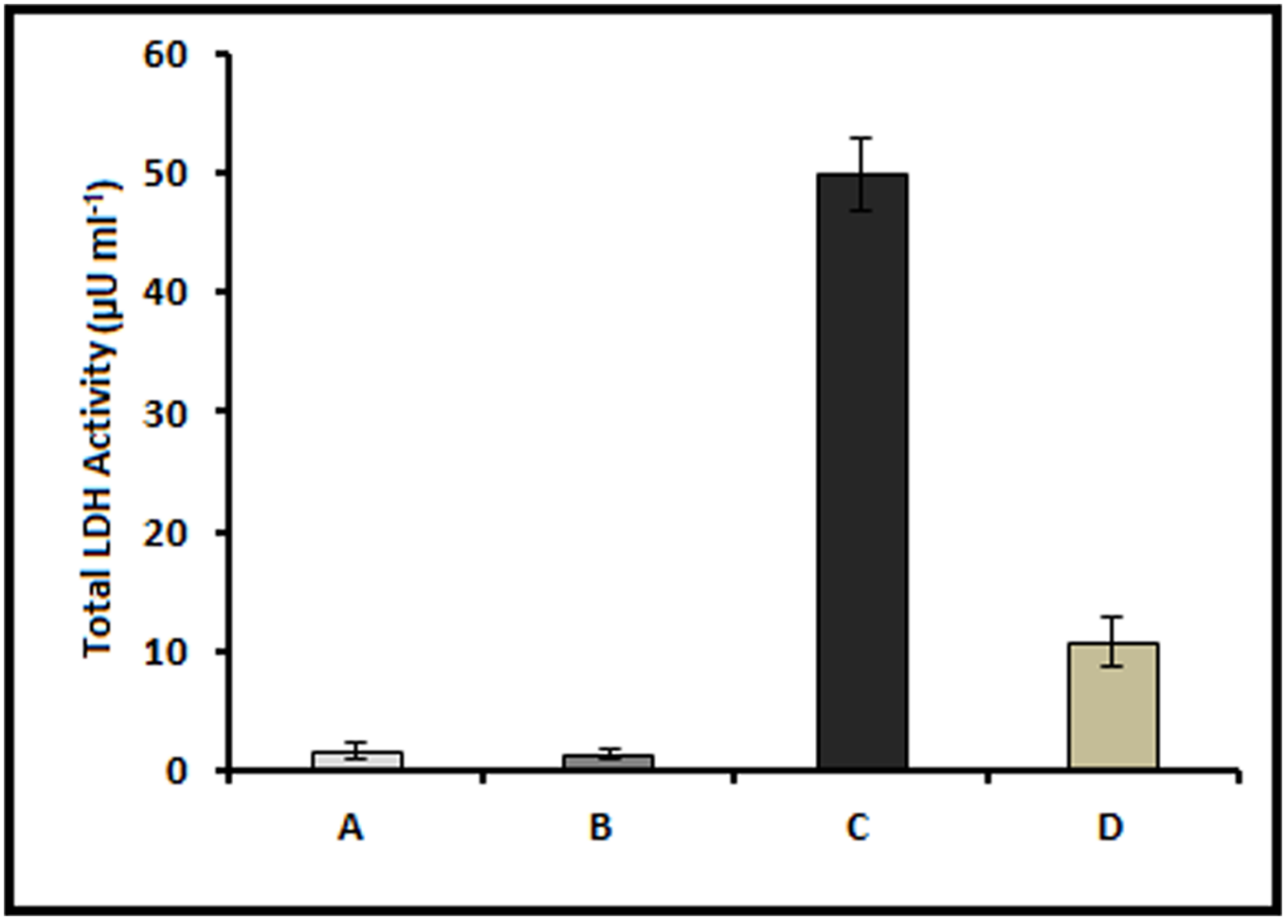
LDH activity in the culture medium of HepG2 cells. LDH assay was performed after 4 h treatment with: (A) only water; (B) only 400 µg ml^−1^ of GLE; (C) only *C. violaceum*; and (D) *C. violaceum* along with 400 µg ml^−1^ GLE.

To analyze the effect of GLE on cell death induced by *C. violaecum*, human hepatocellular liver carcinoma cell line HepG2 was infected with the bacteria either in the absence or presence of GLE. The course of infection was monitored by phase contrast microscopy at different time points. It was observed that *C. violaecum* was able to induce cytotoxicity in HepG2 cells, characterized by shrinkage of the cells and reduction in cell density, with time (Figure. 4A). Whereas, no morphological changes were observed in the cells when infection was carried out in presence of GLE indicating that the extract could protect the HepG2 cells from *C. violaecum* infection (Figure 4B). Notably, GLE alone had no inhibitory effect on the growth of HepG2 cells (Figure 4C). Furthermore, *C. violaceum*-induced cytotoxicity was quantified by assaying the release of the cytosolic enzyme lactate dehydrogenase (LDH). The cell death observed after *C. violaceum* infection in hepatocytes is characterized by membrane rupture with subsequent release of LDH [62]. In agreement, after 4 h following *C. violaceum* infection of HepG2 cells, a significant increase in extracellular LDH activity was observed as compared to the non-infected cells (Fig. 5). Interestingly, the amount of LDH release from *C. violaceum*-infected HepG2 cells decreased by ∼80% in the presence of GLE indicating the ability of GLE to hinder cell death instigated by *C. violaceum*. A similar decrease in cytotoxicity in different cell lines was demonstrated using mutant strains of *C. violaceum* [62]. Moreover, GLE itself had no effect on human hepatoma HepG2 cells showing its non-toxicity towards the host cell (Figure 5). The guava leaf paste was highly likely to be non-toxic since it is used immensely in folklore practices, including as toothpaste for the cure of dental caries [70]. This result shows that GLE regulates the pathogenicity of *C. violaceum* by preventing the initiation of the cascade of gene expression required for successful infection and establishment in the host which, in turn, may provide time to the immune system of the host to eliminate the pathogen. This effect of GLE may be attributed to its ability to either directly or indirectly inhibit the QS system.

## Conclusions

Identification of QS inhibitors (QSIs) from natural products as an alternative to antibiotics is currently an area of intense interest. However, establishment of a real QSI and the phenomenon of resistance to QSIs are debatable aspects in this field of research [71]. The present study proposes guava leaf aqueous extract (GLE) as QSI since it inhibits quorum regulated phenotypes such as violacein production and swarming in the pathogenic bacteria *C. violaceum* and *P. aeruginosa* respectively and induces a global differential gene expression in *C. violaceum*, a pathogen with high mortality rate, without affecting its growth. The complexity of GLE prevents us from directly linking it with the QS system. However, reduction in QS gene expression was correlated to the attenuation of bacterial virulence resulting in prevention of *C. violaceum* induced lysis of host (HepG2) cells *in vitro*. More studies are required to establish the exact nature of the metabolic cross-talks in presence of GLE but this study provides with the platform to think on using the crude extract of plants to combat pathogenic bacteria. Overall, our results provide insights into the candidature of GLE as QSI and the identification of a set of probable quorum regulated genes in *C. violaceum*.

## Supporting Information

Figure S1 **Inhibition of QS-regulated phenotypes by GLE.** **A**. Formation of a colourless translucent zone around the well containing GLE indicating absence of violacein production by *C. violaceum* cells. **B**. Inhibition of swarming motility of *P. aeruginosa* cells grown in presence of GLE.
(TIF)

Table S1 **Significantly down-regulated genes of** ***C. violaceum*** **when grown in presence of GLE.**
(DOCX)

File S1 **Method: Whole genome transcriptome analysis.**
(DOCX)
