# Supplementary figures and images for: Guava Leaf Extract Inhibits Quorum-Sensing and Chromobacterium violaceum Induced Lysis of Human Hepatoma Cells: Whole Transcriptome Analysis Reveals Differential Gene Expression
Source: PLoS One. 2014 Sep 17;9(9):e107703. doi: 10.1371/journal.pone.0107703 (PMC4167859; doi:10.1371/journal.pone.0107703)

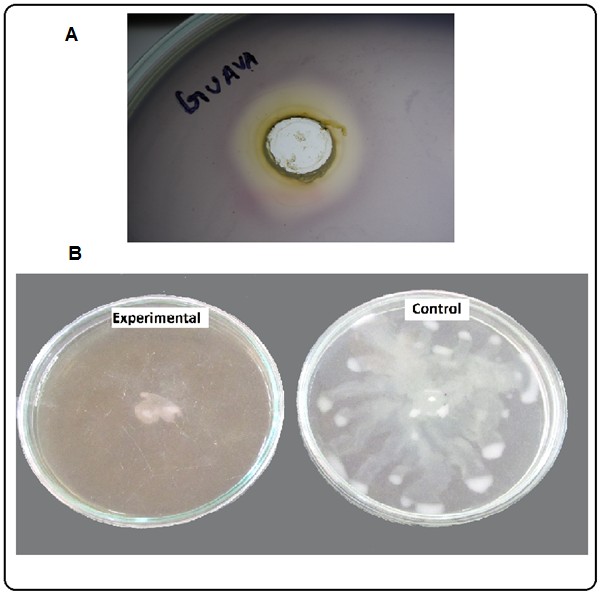

Supplement: Figure S1 — Inhibition of QS-regulated phenotypes by GLE. A. Formation of a colourless translucent zone around the well containing GLE indicating absence of violacein production by C. violaceum cells. B. Inhibition of swarming motility of P. aeruginosa cells grown in presence of GLE. (TIF) [file pone.0107703.s001.tif]
