# Supplementary material for: Guava Leaf Extract Inhibits Quorum-Sensing and Chromobacterium violaceum Induced Lysis of Human Hepatoma Cells: Whole Transcriptome Analysis Reveals Differential Gene Expression
Source: PLoS One. 2014 Sep 17;9(9):e107703. doi: 10.1371/journal.pone.0107703 (PMC4167859; doi:10.1371/journal.pone.0107703)
Supplement: Table S1 — Significantly down-regulated genes of C. violaceum when grown in presence of GLE. (DOCX) [file pone.0107703.s002.docx]

**Table S1**.

| Gene Number | Gene | Description | Control^1^ | Experiment^2^ | log_2_fold | p_value | COG Category^3^ |
| --- | --- | --- | --- | --- | --- | --- | --- |
| CV_1137 | *adhE* | acetaldehyde dehydrogenase | 80.1 | 6.83 | -3.55 | 3.09E-006 | C |
| CV_2470 | *acnB* | aconitate hydratase | 75.94 | 8.38 | -3.18 | 2.56E-005 | C |
| CV_2054 | *acnA2* | aconitate hydratase | 101.32 | 1 | -6.67 | 4.17E-011 | C |
| CV_3282 | *adhC* | alcohol dehydrogenase class III | 74.09 | 8.93 | -3.05 | 4.58E-006 | C |
| CV_0393 | *aldB* | aldehyde dehydrogenase (NAD) | 12.72 | 0.61 | -4.37 | 0 | C |
| CV_2056 | *prpC* | citrate synthase 2 | 280.97 | 5.07 | -5.79 | 6.98E-011 | C |
| CV_3543 |  | conserved hypothetical protein | 18.15 | 1.09 | -4.05 | 0.01 | C |
| CV_0601 | *ctaG* | cytochrome C oxidase assembly transmembrane protein | 511.35 | 2.34 | -7.77 | 2.38E-007 | C |
| CV_0600 | *coxA* | cytochrome-c oxidase, subunit I | 30.97 | 1.11 | -4.8 | 2.46E-005 | C |
| CV_0599 | *coxB* | cytochrome-c oxidase, subunit II | 44.28 | 1.75 | -4.66 | 4.06E-005 | C |
| CV_0603 | *coxC* | cytochrome-c oxidase, subunit III | 76.84 | 7.31 | -3.39 | 6.31E-005 | C |
| CV_0528 | *lpdA1* | dihydrolipoamide dehydrogenase | 11.22 | 1 | -3.49 | 0 | C |
| CV_0527 | *aceF* | dihydrolipoamide S-acetyltransferase | 8.62 | 0.55 | -3.98 | 0.01 | C |
| CV_1412 | *pflB* | formate C-acetyltransferase | 677.66 | 38.39 | -4.14 | 7.50E-007 | C |
| CV_3839 | *fdnG* | formate dehydrogenase | 27.91 | 1.12 | -4.64 | 2.21E-007 | C |
| CV_3369 | *frdA* | fumarate reductase flavoprotein subunit | 127.92 | 15.39 | -3.05 | 5.69E-005 | C |
| CV_3368 | *frdB* | fumarate reductase, subunit B | 127.92 | 15.39 | -3.05 | 5.69E-005 | C |
| CV_3367 | *frdC* | fumarate reductase, subunit C | 127.92 | 15.39 | -3.05 | 5.69E-005 | C |
| CV_2312 | *manA* | mannose-6-phosphate isomerase | 21.61 | 0.35 | -5.95 | 6.86E-005 | C |
| CV_2085 | *mmsA1* | methylmalonate-semialdehyde dehydrogenase | 8.67 | 0.61 | -3.82 | 0.01 | C |
| CV_0055 | *ppc* | phosphoenolpyruvate carboxylase | 14.1 | 1.28 | -3.46 | 7.73E-005 | C |
| CV_2728 |  | probable alcohol dehydrogenase | 463.48 | 54.06 | -3.1 | 4.78E-005 | C |
| CV_4092 |  | probable aldehyde dehydrogenase | 20.7 | 1.39 | -3.9 | 0 | C |
| CV_1174 |  | probable cytochrome-c oxidase, subunit I | 148.15 | 18.31 | -3.02 | 6.32E-005 | C |
| CV_1173 |  | probable cytochrome-c oxidase, subunit II | 187.73 | 15.47 | -3.6 | 1.08E-005 | C |
| CV_1171 |  | probable cytochrome-c oxidase, subunit III | 682.99 | 51.04 | -3.74 | 9.79E-005 | C |
| CV_3818 |  | probable electron transfer flavoprotein, beta subunit | 153.67 | 16.04 | -3.26 | 3.08E-005 | C |
| CV_3027 |  | probable ferredoxin | 113.73 | 13.68 | -3.06 | 3.57E-009 | C |
| CV_2501 |  | probable ferredoxin 2fe-2s protein | 54.43 | 1.96 | -4.79 | 0 | C |
| CV_3028 |  | probable iron-sulphur protein | 113.73 | 13.68 | -3.06 | 3.57E-009 | C |
| CV_3664 |  | probable isocitrate dehydrogenase (NADP) | 147.06 | 12.18 | -3.59 | 1.85E-006 | C |
| CV_1674 |  | probable isoquinoline 1-oxidoreductase | 7.08 | 0.81 | -3.12 | 0.01 | C |
| CV_3304 | *aceB* | probable malate synthase A (msa or masy protein) | 18.57 | 1.72 | -3.43 | 0 | C |
| CV_1813 |  | probable nitroreductase | 44.32 | 5.06 | -3.13 | 0 | C |
| CV_3030 |  | probable oxidoreductase iron-sulfur subunit | 113.73 | 13.68 | -3.06 | 3.57E-009 | C |
| CV_0526 | *aceE* | pyruvate dehydrogenase | 20.23 | 1.63 | -3.64 | 9.20E-006 | C |
| CV_1068 | *sdhB* | succinate dehydrogenase iron-sulfur protein | 131.48 | 6.28 | -4.39 | 9.66E-008 | C |
| CV_4006 | *petC* | ubiquinol-cytochrome c reductase | 126.02 | 12.78 | -3.3 | 3.34E-005 | C |
| CV_3676 |  | probable acetyl-CoA synthetase | 26.3 | 2.26 | -3.54 | 2.00E-006 | C |
| CV_3881 |  | conserved hypothetical protein | 41.85 | 3.8 | -3.46 | 3.10E-005 | C |
| CV_3789 |  | probable glycerate dehydrogenase | 17.71 | 1.07 | -4.05 | 0.01 | C |
| CV_3375 | *minC* | Cell division inhibitor | 40.72 | 1.53 | -4.73 | 0 | D |
| CV_4344 | *ftsW* | cell division protein ftsW | 25.93 | 3.05 | -3.09 | 0 | D |
| CV_4338 | *ftsZ* | cell division protein ftsZ | 61.92 | 7.3 | -3.08 | 9.58E-005 | D |
| CV_0475 | *soj* | chromosome partitioning protein ParA | 25.37 | 1.39 | -4.19 | 0.01 | D |
| CV_0663 | *parA* | chromosome partitioning protein, ParA family ATPase | 111.36 | 12.62 | -3.14 | 2.53E-008 | D |
| CV_4350 |  | conserved hypothetical protein | 157.11 | 0.52 | -8.25 | 3.40E-008 | D |
| CV_1023 | *fleN* | flagellar synthesis regulator FleN | 84.06 | 3.76 | -4.48 | 1.23E-009 | D |
| CV_0661 | *gidA* | glucose inhibited division protein A | 111.36 | 12.62 | -3.14 | 2.53E-008 | D |
| CV_0861 |  | hypothetical protein | 9.01 | 0.31 | -4.88 | 0 | D |
| CV_2971 |  | hypothetical protein | 20.73 | 1.14 | -4.19 | 0.01 | D |
| CV_0833 | *pilM* | type 4 fimbrial biogenesis protein PilM | 130.37 | 15.8 | -3.04 | 5.79E-005 | D |
| CV_0490 | *speB* | agmatinase | 15.57 | 1.04 | -3.9 | 0.01 | E |
| CV_2876 | *speA* | arginine decarboxylase | 7.12 | 0.48 | -3.9 | 0.01 | E |
| CV_1481 | *aroF* | 2-dehydro-3-deoxy-phosphoheptonate aldolase | 38.86 | 4.42 | -3.14 | 0 | E |
| CV_0595 | *leuA* | 2-isopropylmalate synthase | 149.93 | 4.79 | -4.97 | 4.26E-009 | E |
| CV_2784 | *leuC1* | 3-isopropylmalate dehydratase, large subunit | 341.69 | 3.29 | -6.7 | 8.88E-015 | E |
| CV_2782 | *leuD2* | 3-isopropylmalate dehydratase, small subunit | 210.81 | 14.63 | -3.85 | 3.15E-006 | E |
| CV_0966 | *metF* | 5,10-methylenetetrahydrofolate reductase | 73.11 | 7.55 | -3.27 | 0 | E |
| CV_0117 | *gltL* | ABC superfamily (glutamate/aspartate transporter), ATP-binding protein | 36.07 | 4.5 | -3 | 0 | E |
| CV_3921 | *argB* | acetylglutamate kinase | 32.77 | 3.59 | -3.19 | 0 | E |
| CV_2379 | *argE* | acetylornithine deacetylase | 62.77 | 4.16 | -3.92 | 2.08E-006 | E |
| CV_1496 | *aruC* | acetylornithine transaminase | 47.69 | 1.64 | -4.86 | 1.94E-005 | E |
| CV_3735 | *tesA* | acyl-CoA thioesterase | 48.65 | 6.07 | -3 | 0 | E |
| CV_3782 | *arcA* | arginine deiminase | 363.66 | 10.09 | -5.17 | 5.80E-011 | E |
| CV_3783 | *arcD* | arginine/ornithine antiporter | 113.16 | 3.93 | -4.85 | 4.04E-008 | E |
| CV_0115 | *argH* | argininosuccinate lyase | 63.66 | 4.75 | -3.74 | 7.87E-006 | E |
| CV_2382 | *tyrB1* | aromatic-amino-acid transaminase | 82.37 | 7.15 | -3.53 | 1.10E-005 | E |
| CV_0331 | *tyrB2* | aromatic-amino-acid transaminase | 46.83 | 1.61 | -4.86 | 1.94E-005 | E |
| CV_4112 | *aspA* | aspartate ammonia-lyase | 1008.79 | 107.81 | -3.23 | 0 | E |
| CV_2767 | *usg* | aspartate-semialdehyde dehydrogenase | 41.82 | 1.98 | -4.4 | 0 | E |
| CV_3780 | *arcC* | carbamate kinase | 158.03 | 5.38 | -4.88 | 1.03E-008 | E |
| CV_2355 | *pheA* | chorismate mutase/prephenate dehydratase | 82.94 | 10.09 | -3.04 | 8.90E-005 | E |
| CV_0057 | *lasB* | class 4 metalloprotease | 211.37 | 20.93 | -3.34 | 1.09E-006 | E |
| CV_2017 |  | conserved hypothetical protein | 32.38 | 2.38 | -3.77 | 0 | E |
| CV_2018 |  | Conserved hypothetical protein | 32.38 | 2.38 | -3.77 | 0 | E |
| CV_3182 |  | conserved hypothetical protein | 40.6 | 4.45 | -3.19 | 0 | E |
| CV_3361 |  | conserved hypothetical protein | 43.85 | 5.29 | -3.05 | 0.01 | E |
| CV_3613 |  | conserved hypothetical protein | 79.85 | 6.63 | -3.59 | 7.55E-006 | E |
| CV_4074 |  | conserved hypothetical protein | 38.18 | 3.68 | -3.37 | 0 | E |
| CV_4049 | *metB* | cystathionine gamma-synthase | 12.65 | 0.85 | -3.9 | 0.01 | E |
| CV_1795 | *dapB* | dihydrodipicolinate reductase | 87.59 | 7.03 | -3.64 | 0 | E |
| CV_0119 | *gltJ* | glutamate/aspartate transport system permease | 36.34 | 4.45 | -3.03 | 0 | E |
| CV_0118 | *gltK* | glutamate/aspartate transport system permease protein | 36.34 | 4.45 | -3.03 | 0 | E |
| CV_1273 | *glnP* | glutamine transport system permease protein | 29.24 | 3.14 | -3.22 | 0 | E |
| CV_3651 | *glnH* | glutamine transport system substrate-binding protein | 26.42 | 3.03 | -3.12 | 0.01 | E |
| CV_3430 | *gcvH* | glycine cleavage system H protein | 142.76 | 4.3 | -5.05 | 0 | E |
| CV_3429 | *gcvP* | glycine cleavage system P protein | 48.59 | 3.02 | -4.01 | 6.23E-007 | E |
| CV_3431 | *gcvT* | glycine cleavage system T protein | 85.28 | 6.31 | -3.76 | 7.39E-006 | E |
| CV_1286 | *glyA* | glycine hydroxymethyltransferase | 250.6 | 26 | -3.27 | 1.45E-005 | E |
| CV_0854 | *hisM1* | histidine transport system membrane protein M | 27.83 | 1.56 | -4.16 | 0.01 | E |
| CV_0853 | *hisQ1* | histidine transport system permease protein | 27.83 | 1.56 | -4.16 | 0.01 | E |
| CV_0613 | *hisC* | histidinol-phosphate aminotransferase | 30.54 | 3.68 | -3.05 | 0 | E |
| CV_1682 | *hcnC* | hydrogen cyanide synthase HcnC | 187.54 | 6.8 | -4.78 | 7.43E-009 | E |
| CV_2874 | *sdaA1* | L-serine dehydratase | 38.19 | 2.67 | -3.84 | 1.28E-005 | E |
| CV_1408 | *sdaA2* | L-serine dehydratase | 23.25 | 2.67 | -3.12 | 0 | E |
| CV_2045 | *glnB* | nitrogen regulatory protein P-II-1 | 334.25 | 40.31 | -3.05 | 0 | E |
| CV_3781 | *arcB* | ornithine carbamoyltransferase | 274.72 | 6.92 | -5.31 | 8.84E-010 | E |
| CV_2725 | *metZ* | o-succinylhomoserine sulfhydrylase | 36.45 | 2.44 | -3.9 | 5.71E-005 | E |
| CV_3180 | *phhA* | phenylalanine 4-monooxygenase | 40.26 | 3.47 | -3.54 | 0 | E |
| CV_0173 | *aroG* | phospho-2-dehydro-3-deoxyheptonate aldolase | 54.19 | 2.8 | -4.27 | 1.13E-005 | E |
| CV_0621 | *hisE* | phosphoribosyl-ATP pyrophosphohydrolase | 369.42 | 37.65 | -3.29 | 9.97E-005 | E |
| CV_4100 | *potC* | polyamine transport protein PotC | 24.47 | 1.4 | -4.12 | 0.01 | E |
| CV_2833 | *aroB* | probable 3-dehydroquinate synthase | 109.04 | 12.25 | -3.15 | 2.53E-006 | E |
| CV_0792 |  | probable ABC transporter, periplasmic binding protein | 23.56 | 1.42 | -4.05 | 0.01 | E |
| CV_2481 |  | probable amino acid ABC transporter | 263.06 | 18.32 | -3.84 | 3.97E-007 | E |
| CV_3296 |  | probable amino-acid transporter transmembrane protein | 12.76 | 1.34 | -3.25 | 0 | E |
| CV_0224 |  | probable carboxycyclohexadienyl dehydratase | 97.67 | 10.36 | -3.24 | 2.35E-006 | E |
| CV_2804 |  | probable diaminobutyrate-pyruvate transaminase | 13.53 | 1.48 | -3.19 | 0 | E |
| CV_0038 |  | probable histidinol-phosphate aminotransferase | 17.09 | 1.79 | -3.25 | 0 | E |
| CV_2796 |  | probable peptidase, M24 family protein | 14.26 | 1.52 | -3.23 | 0 | E |
| CV_1274 |  | probable permease of ABC transporter | 29.24 | 3.14 | -3.22 | 0 | E |
| CV_2839 |  | probable transporter transmembrane protein | 55.76 | 1.92 | -4.86 | 0 | E |
| CV_3502 | *ptrB* | prolyl oligopeptidase family protein | 7.46 | 0.86 | -3.12 | 0.01 | E |
| CV_1409 | *sdaC* | serine transporter | 91.95 | 8.75 | -3.39 | 1.24E-005 | E |
| CV_0828 | *aroK* | shikimate kinase | 109.04 | 12.25 | -3.15 | 2.53E-006 | E |
| CV_0852 | *argT* | subunit of the lysine/arginine/ornithine transporter (ABC superfamily) | 129.64 | 5.53 | -4.55 | 3.46E-007 | E |
| CV_1456 | *dapE* | succinyl-diaminopimelate desuccinylase | 59.85 | 4.29 | -3.8 | 5.59E-007 | E |
| CV_2454 | *pepQ* | X-Pro dipeptidase | 19.05 | 2.38 | -3 | 0 | E |
| CV_1277 | *ilvD* | dihydroxy-acid dehydratase | 23.26 | 2.42 | -3.27 | 6.13E-005 | E |
| CV_2179 | *trpE* | anthranilate synthase component I | 58.24 | 3.79 | -3.94 | 4.53E-006 | E |
| CV_2094 | *ilvE* | branched-chain-amino-acid transaminase | 209.48 | 22.15 | -3.24 | 1.92E-005 | E |
| CV_0568 |  | probable anthranilate synthase | 41.49 | 0.71 | -5.87 | 8.15E-005 | E |
| CV_3578 | *dapA* | dihydrodipicolinate synthase | 32.47 | 3.56 | -3.19 | 0 | E |
| CV_2051 |  | probable zinc-containing alcohol dehydrogenase | 523.97 | 18.28 | -4.84 | 2.57E-010 | E |
| CV_0369 | *pyrB* | aspartate carbamoyltransferase catalytic chain | 111.65 | 13.57 | -3.04 | 6.92E-005 | F |
| CV_3047 | *cmk* | cytidylate kinase | 51.1 | 3.42 | -3.9 | 0 | F |
| CV_3551 | *pydA* | dihydroorotate oxidase | 64.65 | 2.14 | -4.92 | 1.55E-005 | F |
| CV_1926 | *purU* | formyltetrahydrofolate deformylase | 39.72 | 4.79 | -3.05 | 0 | F |
| CV_0547 | *purD* | phosphoribosylamine-glycine ligase | 15.42 | 1.49 | -3.37 | 0 | F |
| CV_2412 | *nrdD* | ribonucleoside-triphosphate reductase | 28.1 | 2.48 | -3.5 | 3.77E-005 | F |
| CV_1027 | *thyA* | thymidylate synthase | 119.66 | 12.13 | -3.3 | 2.49E-006 | F |
| CV_2198 | *pyrH* | uridylate kinase | 40.96 | 4.63 | -3.15 | 0 | F |
| CV_0622 |  | probable HIT family protein | 202.2 | 12.83 | -3.98 | 0 | F |
| CV_3299 | *treC* | a,a-phosphotrehalase | 8.82 | 0.55 | -4 | 0.01 | G |
| CV_2057 | *prpB* | carboxyvinyl-carboxyphosphonate phosphorylmutase | 202.81 | 8.27 | -4.62 | 8.26E-008 | G |
| CV_2676 | *bscZ* | endo-1,4-D-glucanase | 9.3 | 0.22 | -5.39 | 0 | G |
| CV_2369 | *pgi1* | glucose-6-phosphate isomerase | 102.37 | 9.17 | -3.48 | 2.66E-008 | G |
| CV_0560 | *gapA* | glyceraldehyde-3-phosphate dehydrogenase | 238.07 | 23.08 | -3.37 | 8.64E-006 | G |
| CV_2434 |  | hypothetical protein | 56.1 | 1.93 | -4.86 | 0 | G |
| CV_1062 | *mdh* | malate dehydrogenase | 206.09 | 19.5 | -3.4 | 8.12E-006 | G |
| CV_0189 | *pgk* | phosphoglycerate kinase | 17.03 | 1.64 | -3.37 | 0 | G |
| CV_0357 | *lpcA* | phosphoheptose isomerase | 29.41 | 2.09 | -3.82 | 0.01 | G |
| CV_2172 | *algC* | phosphomannomutase | 49.14 | 4.08 | -3.59 | 2.52E-005 | G |
| CV_4240 |  | probable chitinase | 42.51 | 2.82 | -3.92 | 2.08E-006 | G |
| CV_0816 |  | probable phosphoenolpyruvate-protein phosphotransferase | 31.87 | 3.07 | -3.37 | 6.90E-005 | G |
| CV_3300 | *treB* | protein-N p-phosphohistidine-sugar phosphotransferase | 8.82 | 0.55 | -4 | 0.01 | G |
| CV_0249 | *pykF* | pyruvate kinase | 48.47 | 3.32 | -3.87 | 2.71E-006 | G |
| CV_2311 | *ptsA* | phosphoenolpyruvate-protein phosphotransferase | 21.61 | 0.35 | -5.95 | 6.86E-005 | G |
| CV_0639 |  | hypothetical protein | 12.9 | 0.77 | -4.07 | 0.01 | G |
| CV_3600 | *mhpT* | 3-hydroxyphenylpropionic acid transporter | 26.7 | 0.71 | -5.23 | 0 | G |
| CV_3790 |  | conserved hypothetical protein | 27.25 | 1.37 | -4.32 | 0 | G |
| CV_2958 | *dgoT* | D-galactonate transporter | 11.4 | 0.72 | -3.98 | 0.01 | G |
| CV_3524 |  | probable multidrug efflux protein | 18.12 | 1.37 | -3.73 | 0 | G |
| CV_2237 |  | probable POT family transport protein | 22.2 | 0.74 | -4.9 | 0 | G |
| CV_0336 |  | conserved hypothetical protein | 94.4 | 8.56 | -3.46 | 6.13E-007 | G |
| CV_1361 |  | conserved hypothetical protein | 17.19 | 1.22 | -3.82 | 0.01 | G |
| CV_3334 |  | probable PTS system, fructose-specific IIABC componen | 78.9 | 3.07 | -4.68 | 0 | G |
| CV_2819 |  | probable glutamate-1-semialdehyde aminotransferase | 21.74 | 1.38 | -3.98 | 0 | H |
| CV_4380 | *bioF* | 8-amino-7-oxononanoate synthase | 20.95 | 0.84 | -4.64 | 0 | H |
| CV_0965 | *ahcY* | adenosylhomocysteinase | 34.87 | 2 | -4.12 | 2.20E-005 | H |
| CV_1566 | *cbiD* | cobalamin biosynthesis cbiD transmembrane protein | 59.37 | 2.77 | -4.42 | 5.46E-006 | H |
| CV_3767 |  | conserved hypothetical protein | 210.01 | 5.73 | -5.2 | 8.37E-013 | H |
| CV_1290 | *ribD* | diaminohydroxyphosphoribosylaminopyrimidine deaminase | 26.68 | 2.65 | -3.33 | 0 | H |
| CV_1028 | *folA* | dihydrofolate reductase | 119.66 | 12.13 | -3.3 | 2.49E-006 | H |
| CV_2691 | *ispA* | geranyltranstransferase | 41.68 | 1.15 | -5.18 | 0 | H |
| CV_1650 | *kbl* | glycine C-acetyltransferase | 53.19 | 2.4 | -4.47 | 4.73E-006 | H |
| CV_2361 | *mtrA* | GTP cyclohydrolase I | 54.69 | 4.82 | -3.5 | 0 | H |
| CV_0151 | *thiD* | hydroxymethylpyrimidine kinase | 105.84 | 4.97 | -4.41 | 1.71E-007 | H |
| CV_0884 |  | hypothetical protein | 71.17 | 1.59 | -5.49 | 0 | H |
| CV_1925 | *folD* | methylenetetrahydrofolate dehydrogenase/cyclohydrolase | 198.97 | 13.53 | -3.88 | 1.27E-006 | H |
| CV_3518 | *moaA1* | molybdenum cofactor biosynthesis protein A | 38.62 | 3.1 | -3.64 | 0 | H |
| CV_0185 | *moaE* | molybdopterin converting factor subunit 2 | 26.93 | 1.37 | -4.3 | 0 | H |
| CV_0186 | *moaD* | molybdopterin-converting factor subunit 1 | 200.31 | 12.72 | -3.98 | 0.01 | H |
| CV_3648 | *hemN* | oxygen-independent coproporphyrinogen III oxidase | 76.01 | 6.64 | -3.52 | 8.53E-006 | H |
| CV_1648 | *hemB* | porphobilinogen synthase | 142.4 | 12.9 | -3.46 | 8.19E-006 | H |
| CV_1563 | *cbiG* | precorrin methylase protein | 59.37 | 2.77 | -4.42 | 5.46E-006 | H |
| CV_1565 | *cbiL* | precorrin-2 C20-methyltransferase | 59.37 | 2.77 | -4.42 | 5.46E-006 | H |
| CV_1562 | *cbiJ* | precorrin-3B C17-methyltransferase | 9.36 | 0.6 | -3.97 | 0.01 | H |
| CV_1564 | *cbiF* | precorrin-4 C11-methyltransferase protein | 59.37 | 2.77 | -4.42 | 5.46E-006 | H |
| CV_1568 | *cobL* | precorrin-6Y methylase methyltransferase protein | 59.37 | 2.77 | -4.42 | 5.46E-006 | H |
| CV_1436 |  | probable b-alanine-pyruvate transaminase | 11.07 | 0.7 | -3.98 | 0.01 | H |
| CV_0150 |  | probable thiamine-phosphate diphosphorylase | 105.84 | 4.97 | -4.41 | 1.71E-007 | H |
| CV_0607 | *ctaB* | protoheme IX farnesyltransferase | 153.01 | 12.07 | -3.66 | 5.27E-009 | H |
| CV_1653 | *hemG* | protoporphyrinogen oxidase | 15.23 | 1.41 | -3.43 | 0 | H |
| CV_3678 | *nadA* | quinolinate synthetase | 53.73 | 3.35 | -4 | 3.70E-005 | H |
| CV_3570 | *ribF* | riboflavin kinase | 105.78 | 11.97 | -3.14 | 1.04E-007 | H |
| CV_0294 | *sir2* | transcriptional regulator | 26.12 | 1.5 | -4.12 | 0.01 | H |
| CV_1122 | *hemE* | uroporphyrinogen decarboxylase | 25.07 | 3.02 | -3.05 | 0 | H |
| CV_3886 |  | probable Fe-S oxidoreductase | 79.25 | 6.89 | -3.52 | 2.31E-005 | H |
| CV_3120 | *visC* | oxidoreductase protein | 14.67 | 0.84 | -4.12 | 0.01 | H |
| CV_3269 |  | conserved hypothetical protein | 8.53 | 0.99 | -3.11 | 0.01 | H |
| CV_0906 | *phaJ* | phenylacetic acid permease | 38.97 | 4.46 | -3.13 | 0.01 | H |
| CV_3281 |  | probable sodium:solute symporter | 61.03 | 4.07 | -3.91 | 9.02E-005 | H |
| CV_2692 | *dxs* | 1-deoxy-D-xylulose 5-phosphate synthase | 11.67 | 1.46 | -3 | 0 | H |
| CV_1485 | *entC* | isochorismate synthase EntC / MenF | 480.25 | 29.46 | -4.03 | 8.44E-014 | H |
| CV_1785 |  | probable acyl-CoA dehydrogenase | 30.19 | 2.02 | -3.9 | 9.43E-006 | I |
| CV_2207 | *fabZ* | (3R)-hydroxymyristol acyl carrier protein dehydratase | 41.56 | 4.12 | -3.33 | 0.01 | I |
| CV_2016 | *acpD* | [acyl-carrier-protein] phosphodiesterase | 133.51 | 1.96 | -6.09 | 6.93E-005 | I |
| CV_3282 | *acsA* | acetyl-coenzyme A synthetase | 40.67 | 3.63 | -3.49 | 1.90E-005 | I |
| CV_1111 | *pssA* | CDPdiacylglycerol-serine O-phosphatidyltransferase | 17.48 | 0.7 | -4.64 | 0 | I |
| CV_2371 |  | conserved hypothetical protein | 102.37 | 9.17 | -3.48 | 2.66E-008 | I |
| CV_1758 |  | probable acetyl-coenzyme A synthetase | 18.86 | 0.91 | -4.37 | 0 | I |
| CV_1553 |  | probable enoyl-CoA hydratase | 26.25 | 1.38 | -4.25 | 0.01 | I |
| CV_3062 |  | probable enoyl-CoA hydratase | 32.72 | 1.31 | -4.64 | 0 | I |
| CV_0167 |  | probable lysophospholipase L2 | 25.68 | 2.14 | -3.59 | 0 | I |
| CV_0360 |  | probable thermolabile hemolysin | 25.86 | 2.28 | -3.5 | 0 | I |
| CV_3413 | *acpP* | acyl carrier protein | 875.32 | 39.1 | -4.48 | 4.87E-007 | I |
| CV_0898 | *alkK* | acyl-CoA synthetase | 78.61 | 5.61 | -3.81 | 1.90E-006 | I |
| CV_4163 | *rpsM* | 30S ribosomal protein S13 | 143.13 | 9.86 | -3.86 | 0 | J |
| CV_4057 | *rplY* | 50S ribosomal protein L25 | 583.87 | 34.78 | -4.07 | 4.06E-006 | J |
| CV_1962 | *argS* | arginyl-tRNA synthetase | 154.46 | 17.93 | -3.11 | 3.74E-005 | J |
| CV_1251 |  | conserved hypothetical protein | 47.45 | 3.18 | -3.9 | 0.01 | J |
| CV_3529 |  | conserved hypothetical protein | 40.51 | 1.68 | -4.59 | 5.30E-005 | J |
| CV_3799 |  | conserved hypothetical protein | 51.83 | 5.68 | -3.19 | 0 | J |
| CV_0121 | *ksgA* | dimethyladenosine transferase | 22.59 | 2.72 | -3.05 | 0.01 | J |
| CV_1654 | *glyQ* | glycyl-tRNA synthetase, alpha chain | 28.6 | 1.11 | -4.68 | 0 | J |
| CV_3569 | *ileS* | isoleucyl-tRNA synthetase | 105.78 | 11.97 | -3.14 | 1.04E-007 | J |
| CV_1796 | *omlA* | outer membrane lipoprotein OmlA | 87.59 | 7.03 | -3.64 | 0 | J |
| CV_1740 |  | probable acetyltransferases | 42.27 | 2.01 | -4.4 | 0 | J |
| CV_1288 |  | probable ribosomal-protein-serine acetyltransferase | 36.91 | 2.22 | -4.05 | 0.01 | J |
| CV_1289 |  | probable ribosomal-protein-serine acetyltransferase | 26.68 | 2.65 | -3.33 | 0 | J |
| CV_2787 |  | probable ribosome-associated heat shock protein Hsp15 | 133.71 | 11.41 | -3.55 | 4.29E-005 | J |
| CV_1932 |  | probable RNA methyltransferase | 8.4 | 0.6 | -3.82 | 0.01 | J |
| CV_3333 |  | probable sigma-54 modulation protein | 612.63 | 60.06 | -3.35 | 1.95E-005 | J |
| CV_3618 |  | probable sun homolog protein | 36.38 | 4 | -3.19 | 2.28E-005 | J |
| CV_1818 | *rluC* | pseudouridine synthase | 31.22 | 3.51 | -3.15 | 0 | J |
| CV_1463 | *rbfA* | ribosome-binding factor A | 29.22 | 3.34 | -3.13 | 0 | J |
| CV_0988 | *queA* | S-adenosylmethionine:tRNA ribosyltransferase-isomerase | 16.16 | 0.97 | -4.05 | 0.01 | J |
| CV_1349 | *infC* | translation initiation factor IF-3 | 580.33 | 61.37 | -3.24 | 1.92E-005 | J |
| CV_3402 | *trmU* | tRNA (5-methylaminomethyl-2-thiouridylate)-methyltransferase | 14.62 | 1.68 | -3.12 | 0.01 | J |
| CV_1464 | *truB* | tRNA pseudouridine 55 synthase | 29.22 | 3.34 | -3.13 | 0 | J |
| CV_3058 |  | conserved hypothetical protein | 26.5 | 1.84 | -3.85 | 0.01 | J |
| CV_4142 | *hoxX* | hoxX-like protein | 7.99 | 0.53 | -3.9 | 0.01 | J |
| CV_3667 | *cspE* | cold shock transcription regulator protein | 1023 | 24.7 | -5.37 | 0 | K |
| CV_1213 |  | conserved hypothetical protein | 346.06 | 43.35 | -3 | 0 | K |
| CV_4160 | *rpoA* | DNA-directed RNA polymerase (alpha subunit) | 215.48 | 21.47 | -3.33 | 1.20E-005 | K |
| CV_1608 | *hrcA* | heat-inducible transcription repressor transcription regulator protein | 120.92 | 12.3 | -3.3 | 1.94E-005 | K |
| CV_1536 |  | hypothetical protein | 1442.56 | 92.08 | -3.97 | 2.36E-007 | K |
| CV_0585 |  | probable RNA polymerase sigma factor | 169.08 | 19.69 | -3.1 | 1.20E-005 | K |
| CV_2654 |  | probable transcriptional regulator | 27.35 | 1.57 | -4.12 | 0.01 | K |
| CV_0799 |  | probable transcriptional regulator, AraC/XylS family | 25.5 | 1.4 | -4.19 | 0.01 | K |
| CV_1063 |  | probable transcriptional regulator, GntR family | 86 | 3.57 | -4.59 | 2.72E-006 | K |
| CV_2726 |  | probable transcriptional regulator, MarR family | 112.11 | 9.89 | -3.5 | 0 | K |
| CV_0711 |  | probable transcriptional regulator, TetR family | 54.53 | 1.99 | -4.77 | 0 | K |
| CV_2091 |  | probable transcriptional regulator, TetR family | 23.69 | 2.89 | -3.03 | 0.01 | K |
| CV_2456 |  | probable transcriptional regulator, TetR family | 42.02 | 3.9 | -3.43 | 0 | K |
| CV_3037 | *pdhR* | pyruvate dehydrogenase complex repressor | 23.31 | 1.4 | -4.05 | 0.01 | K |
| CV_2066 | *rnc* | ribonuclease III | 123.72 | 10.35 | -3.58 | 9.49E-009 | K |
| CV_3332 | *rpoN* | RNA polymerase N (sigma54) factor | 132.3 | 10.67 | -3.63 | 2.64E-006 | K |
| CV_1022 | *fliA1* | RNA polymerase sigma factor for flagellar operon | 84.06 | 3.76 | -4.48 | 1.23E-009 | K |
| CV_4090 | *cviR* | transcriptional activator, LuxR/UhpA family of regulators. | 194.51 | 14.75 | -3.72 | 3.04E-006 | K |
| CV_3302 | *treR* | transcriptional repressor of the trehalose operon | 40.86 | 4.77 | -3.1 | 0 | K |
| CV_1451 |  | probable transcriptional regulator | 122.76 | 14.86 | -3.05 | 5.33E-005 | K |
| CV_1900 |  | probable transcriptional regulator | 32.64 | 0.62 | -5.71 | 0 | K |
| CV_0112 | *glpR1* | glycerol-3-phosphate regulon repressor | 85.07 | 4.21 | -4.34 | 8.59E-006 | K |
| CV_3388 |  | conserved hypothetical protein | 84.36 | 7.63 | -3.47 | 0 | K |
| CV_1329 | *sbcB* | exodeoxyribonuclease I | 15.82 | 0.66 | -4.59 | 0 | L |
| CV_3869 |  | probable DNA-binding protein hu-beta | 163.97 | 10.4 | -3.98 | 0.01 | L |
| CV_4068 | *rep* | ATP-dependent DNA helicase | 11.31 | 0.88 | -3.68 | 0 | L |
| CV_4249 |  | conserved hypothetical protein | 30.9 | 2.07 | -3.9 | 0.01 | L |
| CV_3720 |  | conserved hypothetical protein | 39.62 | 2.81 | -3.82 | 0 | L |
| CV_3903 | *lig* | DNA ligase | 9.09 | 1.06 | -3.1 | 0 | L |
| CV_2069 | *recO* | DNA repair protein | 123.72 | 10.35 | -3.58 | 9.49E-009 | L |
| CV_3079 | *radC* | DNA repair protein RadC | 56.06 | 6.76 | -3.05 | 0 | L |
| CV_2321 | *recN* | DNA repair protein recN | 19.88 | 0.54 | -5.19 | 0 | L |
| CV_4269 | *topA* | DNA topoisomerase | 35.27 | 2.66 | -3.73 | 8.36E-006 | L |
| CV_1257 | *rnhA* | DNA-directed DNA polymerase, epsilon chain | 158.57 | 9.89 | -4 | 3.71E-005 | L |
| CV_3152 | *uvrB* | excinuclease ABC subunit B | 10.63 | 0.44 | -4.59 | 0 | L |
| CV_1305 | *uvrC* | excinuclease ABC subunit C | 36.09 | 2 | -4.18 | 3.71E-007 | L |
| CV_2475 | *recJ* | exodeoxyribonuclease VII | 8.93 | 1.08 | -3.05 | 0.01 | L |
| CV_2690 | *xseB* | exodeoxyribonuclease VII small subunit | 41.68 | 1.15 | -5.18 | 0 | L |
| CV_2372 | *xerC* | integrase/recombinase XerC | 70.32 | 4.58 | -3.94 | 7.74E-006 | L |
| CV_4101 | *potB* | polyamine transport protein PotB | 34.81 | 2.33 | -3.9 | 0 | L |
| CV_1020 |  | probable DNA-directed DNA polymerase III (epsilon subunit) | 12.8 | 0.64 | -4.32 | 0 | L |
| CV_0958 |  | probable tis1421-transposase orfa protein | 50 | 2.19 | -4.51 | 0.01 | L |
| CV_0957 |  | probable TIS1421-transposase orfB protein | 50 | 2.19 | -4.51 | 0.01 | L |
| CV_2600 |  | probable two-component sensor/regulator | 17.29 | 1.24 | -3.8 | 1.56E-005 | L |
| CV_2210 | *rnhB* | ribonuclease HII | 29.44 | 3.69 | -3 | 0 | L |
| CV_1889 | *ssb* | single-strand DNA-binding protein | 137.3 | 8.9 | -3.95 | 0 | L |
| CV_1113 | *parE* | topoisomerase IV subunit B | 21.05 | 1.36 | -3.95 | 4.59E-005 | L |
| CV_3614 |  | conserved hypothetical protein | 79.85 | 6.63 | -3.59 | 7.55E-006 | L |
| CV_0933 | *recG* | ATP-dependent DNA helicase | 11.86 | 0.87 | -3.78 | 0 | L |
| CV_2747 | *kdsA* | 2-dehydro-3-deoxyphosphooctonate aldolase | 26.14 | 1.24 | -4.4 | 0 | M |
| CV_3344 | *kdsB* | 3-deoxy-manno-octulosonate cytidylyltransferase | 368.3 | 5.77 | -6 | 1.91E-011 | M |
| CV_2092 | *rfaF* | ADP-heptose--LPS heptosyltransferase II | 23.69 | 2.89 | -3.03 | 0.01 | M |
| CV_3793 | *dadB* | alanine racemase | 65.44 | 4.59 | -3.83 | 3.23E-006 | M |
| CV_0674 | *glmU* | bifuncional: UDP-N-acetylglucosamineglucose-1-phosphate thymidylyltransferase; Glucosamine-1-phosphate | 21.63 | 0.69 | -4.98 | 0 | M |
| CV_4340 | *ftsQ* | cell division transmembrane protein | 41.07 | 1.13 | -5.19 | 0 | M |
| CV_0400 |  | conserved hypothetical protein | 379.87 | 30.87 | -3.62 | 1.53E-008 | M |
| CV_0348 |  | conserved hypothetical protein | 10.41 | 0.73 | -3.84 | 0.01 | M |
| CV_0752 |  | conserved hypothetical protein | 10.46 | 1.1 | -3.25 | 0 | M |
| CV_1983 |  | conserved hypothetical protein | 54.04 | 3.57 | -3.92 | 0 | M |
| CV_3606 |  | conserved hypothetical protein | 34.84 | 3.18 | -3.45 | 0 | M |
| CV_3617 |  | conserved hypothetical protein | 36.38 | 4 | -3.19 | 2.28E-005 | M |
| CV_3906 |  | conserved hypothetical protein | 156.81 | 7.39 | -4.41 | 5.66E-008 | M |
| CV_4351 |  | conserved hypothetical protein | 157.11 | 0.52 | -8.25 | 3.40E-008 | M |
| CV_0978 | *cfa* | cyclopropane-fatty-acyl-phospholipid synthase | 38.99 | 4.2 | -3.22 | 7.84E-005 | M |
| CV_4341 | *ddlB* | D-alanine--D-alanine ligase | 41.07 | 1.13 | -5.19 | 0 | M |
| CV_4011 | *rfbD* | dTDP-4-dehydrorhamnose reductase | 16.98 | 0.93 | -4.19 | 0.01 | M |
| CV_4010 | *rmlB* | dTDPglucose 4,6-dehydratase | 16.98 | 0.93 | -4.19 | 0.01 | M |
| CV_0662 | *gidB* | glucose inhibited division protein B | 111.36 | 12.62 | -3.14 | 2.53E-008 | M |
| CV_4012 | *rfbA* | glucose-1-phosphate thymidylyltransferase | 16.98 | 0.93 | -4.19 | 0.01 | M |
| CV_3146 | *hyuA* | hydantoin racemase | 19.31 | 1.47 | -3.71 | 0.01 | M |
| CV_3976 |  | hypothetical protein | 61.67 | 3.38 | -4.19 | 0.01 | M |
| CV_2571 | *lasA* | LasA protease precursor | 175.34 | 13.11 | -3.74 | 1.13E-006 | M |
| CV_2209 | *lpxB* | lipid-A-disaccharide synthase | 29.44 | 3.69 | -3 | 0 | M |
| CV_4033 | *neuC* | N-acetylglucosamine-6-phosphate 2-epimerase/N-acetylglucosamine-6-phosphatase | 91.04 | 10.87 | -3.07 | 2.02E-007 | M |
| CV_2205 | *ompH* | outer membrane protein | 46.62 | 5.62 | -3.05 | 0.01 | M |
| CV_4103 | *ompW* | outer membrane protein W precursor | 243.72 | 28.66 | -3.09 | 5.08E-005 | M |
| CV_3586 | *mtgA* | peptidoglycan glycosyltransferase | 36.9 | 3.3 | -3.49 | 0 | M |
| CV_3759 | *mltA* | peptidoglycan N-acetylmuramoylhydrolase | 29.48 | 2.27 | -3.7 | 0 | M |
| CV_1609 | *mltB* | peptidoglycan N-acetylmuramoylhydrolase | 41.91 | 4.77 | -3.14 | 0 | M |
| CV_2034 | *sltY* | peptidoglycan N-acetylmuramoylhydrolase | 20.06 | 1.9 | -3.4 | 9.93E-005 | M |
| CV_4346 | *mraY* | phospho-N-acetylmuramoyl-pentapeptide-transferase | 19.51 | 1.81 | -3.43 | 0 | M |
| CV_4034 |  | probable aminotransferase | 91.04 | 10.87 | -3.07 | 2.02E-007 | M |
| CV_0750 |  | probable glutamine-scyllo-inositol transaminase | 20.85 | 2.6 | -3 | 0 | M |
| CV_0817 |  | probable glycosyltransferase | 16.92 | 1.7 | -3.32 | 0 | M |
| CV_2871 |  | probable hemagglutinin | 14.84 | 0.71 | -4.38 | 0.01 | M |
| CV_2385 |  | probable integral membrane protein | 27.81 | 2.48 | -3.49 | 0 | M |
| CV_4060 |  | probable outer membrane lipoprotein | 92.22 | 9.61 | -3.26 | 9.85E-006 | M |
| CV_3829 |  | probable outer membrane porin precursor | 813.08 | 69.81 | -3.54 | 6.04E-006 | M |
| CV_0533 |  | probable outer membrane protein | 19.52 | 2.14 | -3.19 | 0 | M |
| CV_1989 |  | probable porin protein | 257.04 | 24.41 | -3.4 | 6.76E-006 | M |
| CV_3104 |  | probable porin signal peptide protein | 1207.93 | 95.97 | -3.65 | 7.68E-006 | M |
| CV_1860 |  | probable transmembrane protein | 21.44 | 1.32 | -4.02 | 1.17E-006 | M |
| CV_2765 |  | probable transmembrane protein | 24.68 | 1.8 | -3.78 | 1.74E-005 | M |
| CV_2766 |  | probable transmembrane protein | 35.67 | 2.32 | -3.94 | 4.53E-006 | M |
| CV_4358 | *mreC* | rod shape-determining protein mreC | 23.46 | 2.26 | -3.37 | 0 | M |
| CV_2206 | *lpxD* | UDP-3-O-[3-hydroxymyristoyl] glucosamine N-acyltransferase (firA protein) | 41.56 | 4.12 | -3.33 | 0.01 | M |
| CV_4129 | *udg* | UDPglucose 6-dehydrogenase | 54.14 | 5.13 | -3.4 | 4.20E-005 | M |
| CV_0440 | *murA* | UDP-N-acetylglucosamine 1-carboxyvinyltransferase | 18.29 | 1.52 | -3.59 | 0 | M |
| CV_4345 | *murD* | UDP-N-acetylmuramoylalanine-D-glutamate ligase | 25.93 | 3.05 | -3.09 | 0 | M |
| CV_3890 |  | probable spore coat polysaccharide biosynthesis protein E | 17.55 | 1.87 | -3.23 | 0 | M |
| CV_2330 |  | conserved hypothetical protein | 19.45 | 0.6 | -5.02 | 0 | M |
| CV_3892 | *rfbG* | CDPglucose 4,6-dehydratase | 48.71 | 5.16 | -3.24 | 7.95E-005 | M |
| CV_2033 |  | probable NADH-ubiquinone oxidoreductase | 20.62 | 2.16 | -3.25 | 0 | M |
| CV_4035 |  | probable nucleotide sugar dehydratase | 78.18 | 1.11 | -6.14 | 6.52E-005 | M |
| CV_3893 | *ddhA* | glucose-1-phosphate cytidylyltransferase | 48.71 | 5.16 | -3.24 | 7.95E-005 | M |
| CV_0465 |  | conserved hypothetical protein | 235.52 | 10.63 | -4.47 | 6.56E-013 | M |
| CV_2242 | *ibeB* | outer membrane drug efflux lipoprotein | 13.9 | 0.27 | -5.67 | 0 | M |
| CV_0433 | *oprM* | outer membrane efflux protein | 55.32 | 4.15 | -3.74 | 1.83E-008 | M |
| CV_0308 |  | probable agglutination protein | 41.89 | 1.97 | -4.41 | 6.08E-006 | M |
| CV_2703 |  | probable outer membrane protein | 25.04 | 1.37 | -4.19 | 0 | M |
| CV_1986 | *exbD4* | biopolymer transport exbD transmembrane protein | 107.73 | 8.12 | -3.73 | 0.01 | N |
| CV_1985 | *exbD1* | biopolymer transport exbD transmembrane protein | 133.98 | 13.18 | -3.35 | 0 | N |
| CV_1974 | *exbD3* | biopolymer transport exbD transmembrane protein | 107.73 | 8.12 | -3.73 | 0.01 | N |
| CV_1973 | *exbD5* | biopolymer transport exbD transmembrane protein | 133.98 | 13.18 | -3.35 | 0 | N |
| CV_0398 | *exbD2* | biopolymer transport exbD transmembrane protein | 379.87 | 30.87 | -3.62 | 1.53E-008 | N |
| CV_2618 | *sipC* | cell invasion protein | 17.62 | 0.92 | -4.25 | 0.01 | N |
| CV_2026 | *motA1* | chemotaxis motA protein | 19.3 | 1.22 | -3.98 | 0.01 | N |
| CV_1814 | *motB1* | chemotaxis motB protein | 44.32 | 5.06 | -3.13 | 0 | N |
| CV_2027 | *motB2* | chemotaxis motB protein | 58.12 | 6.67 | -3.12 | 0 | N |
| CV_3442 | *cheA* | chemotaxis protein CheA | 28.24 | 1.17 | -4.59 | 2.71E-006 | N |
| CV_1014 | *cheA2* | chemotaxis protein CheA | 18.23 | 1.14 | -4 | 0 | N |
| CV_3449 | *cheZ* | chemotaxis protein CheZ | 76.95 | 5.68 | -3.76 | 0 | N |
| CV_3808 |  | conserved hypothetical protein | 75.31 | 8.56 | -3.14 | 0 | N |
| CV_1026 | *flhB1* | flagellar biosynthetic protein flhB | 28.19 | 1.66 | -4.09 | 0 | N |
| CV_1024 | *flhF* | flagellar biosynthetic protein FlhF | 84.06 | 3.76 | -4.48 | 1.23E-009 | N |
| CV_3131 | *fliL* | flagellar fliL transmembrane protein | 242.29 | 9.42 | -4.68 | 1.74E-006 | N |
| CV_2879 | *flgK1* | flagellar hook-associated protein 1 | 88.31 | 7.57 | -3.54 | 4.31E-006 | N |
| CV_3137 | *fliE* | flagellar hook-basal body complex protein | 431.01 | 25.67 | -4.07 | 4.05E-006 | N |
| CV_3130 | *fliM* | flagellar motor switch protein | 257.98 | 20.46 | -3.66 | 5.47E-008 | N |
| CV_3129 | *fliN* | flagellar motor switch protein | 257.98 | 20.46 | -3.66 | 5.47E-008 | N |
| CV_2495 | *fliC2* | flagellin | 35.39 | 2.44 | -3.86 | 0 | N |
| CV_3878 | *fliC3* | flagellin | 750.37 | 66.73 | -3.49 | 4.37E-006 | N |
| CV_3877 | *flaG* | Flagellin protein | 434.03 | 32.04 | -3.76 | 3.46E-006 | N |
| CV_3134 | *fliI1* | flagellum-specific ATP synthase | 16.61 | 0.67 | -4.64 | 0 | N |
| CV_3809 | *gspI* | general secretion pathway protein I | 75.31 | 8.56 | -3.14 | 0 | N |
| CV_3810 | *gspH* | general secretory pathway protein H | 75.31 | 8.56 | -3.14 | 0 | N |
| CV_3807 | *gspK* | general secretory pathway protein K | 75.31 | 8.56 | -3.14 | 0 | N |
| CV_3874 |  | hypothetical protein | 796.91 | 48.05 | -4.05 | 1.13E-006 | N |
| CV_4297 | *trg* | methyl-accepting chemotaxis protein III (MCP-III) | 16.18 | 1.11 | -3.86 | 0 | N |
| CV_1417 | *nahY* | methyl-accepting chemotaxis transducer protein | 18.34 | 1.66 | -3.47 | 0 | N |
| CV_3450 |  | probable chemotaxis protein CheA | 53.89 | 4.24 | -3.67 | 5.43E-006 | N |
| CV_0399 |  | probable exbB-like biopolymer transport | 379.87 | 30.87 | -3.62 | 1.53E-008 | N |
| CV_3348 |  | probable exbB-like biopolymer transport | 32.09 | 1.93 | -4.05 | 0.01 | N |
| CV_3127 |  | probable flagellar biosynthetic protein fliP | 52.76 | 4.35 | -3.6 | 0 | N |
| CV_2305 |  | probable flagellar protein | 50.27 | 0.68 | -6.21 | 5.06E-005 | N |
| CV_3729 |  | probable flagellar protein | 12.88 | 0.6 | -4.43 | 0 | N |
| CV_3876 |  | probable flagellar protein | 241.28 | 28.74 | -3.07 | 4.60E-005 | N |
| CV_3806 |  | probable general secretion pathway protein L | 75.31 | 8.56 | -3.14 | 0 | N |
| CV_2489 |  | probable glucose-methanol-choline oxidoreductase | 9.84 | 1.19 | -3.05 | 0.01 | N |
| CV_0899 |  | probable methyl-accepting chemotaxis protein | 71.58 | 7.6 | -3.24 | 2.16E-005 | N |
| CV_1328 |  | probable methyl-accepting chemotaxis protein | 39.91 | 2.86 | -3.8 | 3.86E-006 | N |
| CV_2859 |  | probable methyl-accepting chemotaxis protein | 15.48 | 1.7 | -3.19 | 0 | N |
| CV_3320 |  | probable methyl-accepting chemotaxis protein | 10.05 | 0.45 | -4.49 | 0 | N |
| CV_3866 |  | probable methyl-accepting chemotaxis protein | 26.05 | 2.8 | -3.22 | 7.84E-005 | N |
| CV_4156 |  | probable methyl-accepting chemotaxis protein | 139.82 | 12.41 | -3.49 | 3.91E-006 | N |
| CV_1013 |  | probable methyl-accepting chemotaxis protein II | 18.23 | 1.14 | -4 | 0 | N |
| CV_0395 |  | probable methyl-accepting chemotaxis protein IV | 17.92 | 1.31 | -3.77 | 0 | N |
| CV_4244 |  | probable methyl-accepting chemotaxis transducer | 13.75 | 1.11 | -3.64 | 0 | N |
| CV_0095 |  | probable methyl-accepting chemotaxis transducer | 14.88 | 0.56 | -4.73 | 0 | N |
| CV_2509 |  | probable methyl-accepting chemotaxis transducer transmembrane protein | 8.17 | 0.55 | -3.9 | 0.01 | N |
| CV_0347 |  | probable phage virion | 10.41 | 0.73 | -3.84 | 0.01 | N |
| CV_0479 |  | probable type-4 fimbrial biogenesis protein | 78.67 | 0.93 | -6.41 | 1.77E-005 | N |
| CV_3111 |  | probable type-4 fimbrial pilin related signal peptide protein | 79.2 | 2.24 | -5.14 | 0 | N |
| CV_1345 | *secD* | protein-export membrane protein secD | 23.39 | 2.92 | -3 | 0 | N |
| CV_3346 | *lpxK* | tetraacyldisaccharide 4`-kinase | 368.3 | 5.77 | -6 | 1.91E-011 | N |
| CV_0180 | *pilU2* | twitching mobility protein transport fimbria | 32.64 | 2.57 | -3.67 | 0 | N |
| CV_1458 | *pilU1* | twitching motility protein | 41.43 | 5.17 | -3 | 0 | N |
| CV_0179 | *pilT* | twitching motility protein PilT | 53.97 | 6.7 | -3.01 | 0 | N |
| CV_3828 | *pilB* | type 4 fimbrial biogenesis protein | 63.95 | 5.32 | -3.59 | 5.99E-006 | N |
| CV_0830 | *pilP* | type 4 fimbrial biogenesis protein PilP | 96.39 | 2.11 | -5.51 | 1.01E-013 | N |
| CV_0829 | *pilQ* | type 4 fimbrial biogenesis protein PilQ | 96.39 | 2.11 | -5.51 | 1.01E-013 | N |
| CV_2602 | *escV* | type III secretion system EscV protein | 19.3 | 0.43 | -5.48 | 0 | N |
| CV_3112 | *pilV* | type-4 fimbrial biogenesis PilV transmembrane protein | 79.2 | 2.24 | -5.14 | 0 | N |
| CV_1080 |  | Unassigned | 16.15 | 1.42 | -3.5 | 0 | N |
| CV_2619 | *sipB* | cell invasion protein | 116.65 | 3.16 | -5.21 | 6.23E-005 |  |
| CV_2218 |  | conserved hypothetical protein | 10.41 | 1.23 | -3.08 | 0 | N |
| CV_3540 |  | probable fimbrial biogenesis and twitching motility protein | 79.43 | 9.57 | -3.05 | 1.09E-006 | N |
| CV_3447 | *cheV1* | chemotaxis protein CheV | 229.49 | 19.45 | -3.56 | 3.32E-006 | N |
| CV_0239 |  | conserved hypothetical protein | 50.88 | 1.73 | -4.88 | 1.80E-005 | N |
| CV_0531 |  | conserved hypothetical protein | 14.58 | 0.8 | -4.19 | 0.01 | N |
| CV_1033 |  | conserved hypothetical protein | 46.83 | 3.37 | -3.79 | 2.03E-006 | N |
| CV_3205 |  | conserved hypothetical protein | 42.52 | 3.53 | -3.59 | 4.25E-005 | N |
| CV_4237 |  | probable GGDEF family protein | 10.97 | 0.63 | -4.12 | 0.01 | N |
| CV_2677 | *bcsB* | cellulose synthase, subunit B | 9.3 | 0.22 | -5.39 | 0 | NRF |
| CV_2222 |  | conserved hypothetical protein | 160.12 | 11.45 | -3.81 | 5.99E-007 | NRF |
| CV_3169 |  | conserved hypothetical protein | 131.61 | 10.07 | -3.71 | 2.40E-005 | NRF |
| CV_0907 |  | hypothetical protein | 38.97 | 4.46 | -3.13 | 0.01 | NRF |
| CV_2272 |  | hypothetical protein | 85.46 | 10.3 | -3.05 | 8.35E-005 | NRF |
| CV_2283 |  | hypothetical protein | 22.4 | 0.52 | -5.43 | 0 | NRF |
| CV_2378 |  | hypothetical protein | 687.15 | 82.88 | -3.05 | 0 | NRF |
| CV_3128 |  | probable Flagellar protein FliO | 52.76 | 4.35 | -3.6 | 0 | NRF |
| CV_2266 |  | probable SPI2 translocated effector | 17.97 | 0.98 | -4.19 | 0.01 | NRF |
| CV_2546 |  | conserved hypothetical protein | 77.79 | 6.98 | -3.48 | 7.20E-006 | NRF |
| CV_2318 |  | hypothetical protein | 74.5 | 4.99 | -3.9 | 0 | NRF |
| CV_1965 |  | probable protease | 11.68 | 0.54 | -4.43 | 0 | NRF |
| CV_0903 |  | conserved hypothetical protein | 38.97 | 4.46 | -3.13 | 0.01 | NRF |
| CV_0223 |  | conserved hypothetical protein | 97.67 | 10.36 | -3.24 | 2.35E-006 | NRF |
| CV_0408 |  | conserved hypothetical protein | 88.68 | 10.54 | -3.07 | 0 | NRF |
| CV_0464 |  | conserved hypothetical protein | 40.86 | 1.19 | -5.1 | 1.72E-009 | NRF |
| CV_0470 |  | conserved hypothetical protein | 53.21 | 4.14 | -3.68 | 0 | NRF |
| CV_0480 |  | conserved hypothetical protein | 78.67 | 0.93 | -6.41 | 1.77E-005 | NRF |
| CV_0605 |  | conserved hypothetical protein | 113.34 | 7.22 | -3.97 | 1.89E-006 | NRF |
| CV_0775 |  | conserved hypothetical protein | 234.02 | 20.16 | -3.54 | 0 | NRF |
| CV_1083 |  | conserved hypothetical protein | 59.41 | 3.26 | -4.19 | 0.01 | NRF |
| CV_1208 |  | conserved hypothetical protein | 1166.09 | 43.95 | -4.73 | 1.40E-009 | NRF |
| CV_1469 |  | conserved hypothetical protein | 10.63 | 0.77 | -3.78 | 0 | NRF |
| CV_1470 |  | conserved hypothetical protein | 10.63 | 0.77 | -3.78 | 0 | NRF |
| CV_1639 |  | conserved hypothetical protein | 62 | 4.27 | -3.86 | 0 | NRF |
| CV_1733 |  | conserved hypothetical protein | 10.75 | 0.65 | -4.05 | 0.01 | NRF |
| CV_1744 |  | conserved hypothetical protein | 669.33 | 5.53 | -6.92 | 7.19E-006 | NRF |
| CV_1751 |  | conserved hypothetical protein | 13.74 | 1.62 | -3.08 | 0.01 | NRF |
| CV_1752 |  | conserved hypothetical protein | 13.74 | 1.62 | -3.08 | 0.01 | NRF |
| CV_1753 |  | conserved hypothetical protein | 13.74 | 1.62 | -3.08 | 0.01 | NRF |
| CV_1754 |  | conserved hypothetical protein | 13.74 | 1.62 | -3.08 | 0.01 | NRF |
| CV_1963 |  | conserved hypothetical protein | 26.61 | 2.42 | -3.46 | 7.73E-005 | NRF |
| CV_2003 |  | conserved hypothetical protein | 52.5 | 3.67 | -3.84 | 1.28E-005 | NRF |
| CV_2090 |  | conserved hypothetical protein | 190.01 | 21.64 | -3.13 | 4.39E-005 | NRF |
| CV_2105 |  | Conserved hypothetical protein | 14.27 | 0.91 | -3.96 | 0.01 | NRF |
| CV_2124 |  | conserved hypothetical protein | 33.87 | 3.51 | -3.27 | 0 | NRF |
| CV_2436 |  | conserved hypothetical protein | 10.54 | 1.21 | -3.12 | 0.01 | NRF |
| CV_2441 |  | conserved hypothetical protein | 33.89 | 2.72 | -3.64 | 0 | NRF |
| CV_2494 |  | conserved hypothetical protein | 21.19 | 1.28 | -4.05 | 0 | NRF |
| CV_2519 |  | conserved hypothetical protein | 140.01 | 10.23 | -3.77 | 0 | NRF |
| CV_2681 |  | conserved hypothetical protein | 146.74 | 6.43 | -4.51 | 6.98E-005 | NRF |
| CV_2780 |  | conserved hypothetical protein | 113.33 | 8.41 | -3.75 | 1.93E-005 | NRF |
| CV_2781 |  | conserved hypothetical protein | 119.19 | 8.21 | -3.86 | 0 | NRF |
| CV_2970 |  | conserved hypothetical protein | 237.8 | 26.07 | -3.19 | 8.92E-005 | NRF |
| CV_3059 |  | conserved hypothetical protein | 26.5 | 1.84 | -3.85 | 0.01 | NRF |
| CV_3268 |  | conserved hypothetical protein | 8.53 | 0.99 | -3.11 | 0.01 | NRF |
| CV_3350 |  | conserved hypothetical protein | 58.54 | 6.14 | -3.25 | 0 | NRF |
| CV_3374 |  | conserved hypothetical protein | 63.01 | 2.17 | -4.86 | 0 | NRF |
| CV_3451 |  | conserved hypothetical protein | 20.13 | 0.78 | -4.68 | 0 | NRF |
| CV_3470 |  | conserved hypothetical protein | 36.13 | 2.78 | -3.7 | 0 | NRF |
| CV_3572 |  | conserved hypothetical protein | 40.05 | 2.84 | -3.82 | 0.01 | NRF |
| CV_3580 |  | conserved hypothetical protein | 4644.42 | 172.92 | -4.75 | 3.78E-008 | NRF |
| CV_3610 |  | conserved hypothetical protein | 73.81 | 3.71 | -4.32 | 0 | NRF |
| CV_3728 |  | conserved hypothetical protein | 316.33 | 21.83 | -3.86 | 8.88E-006 | NRF |
| CV_3824 |  | conserved hypothetical protein | 64.28 | 5.74 | -3.49 | 6.81E-005 | NRF |
| CV_3966 |  | conserved hypothetical protein | 21.1 | 0.95 | -4.47 | 0 | NRF |
| CV_3973 |  | conserved hypothetical protein | 42.61 | 2.79 | -3.93 | 0 | NRF |
| CV_3974 |  | conserved hypothetical protein | 42.61 | 2.79 | -3.93 | 0 | NRF |
| CV_4029 |  | conserved hypothetical protein | 21.53 | 0.95 | -4.51 | 0 | NRF |
| CV_4075 |  | conserved hypothetical protein | 77.08 | 7.48 | -3.36 | 1.42E-005 | NRF |
| CV_4107 |  | conserved hypothetical protein | 22.62 | 0.52 | -5.45 | 0 | NRF |
| CV_4133 |  | conserved hypothetical protein | 12.95 | 1.56 | -3.05 | 0.01 | NRF |
| CV_4296 |  | conserved hypothetical protein | 16.69 | 0.67 | -4.64 | 0 | NRF |
| CV_4304 |  | conserved hypothetical protein | 48.31 | 4.85 | -3.32 | 0 | NRF |
| CV_4333 |  | conserved hypothetical protein | 84.67 | 7.85 | -3.43 | 0 | NRF |
| CV_1262 | *ppx* | exopolyphosphatase | 19.52 | 1.86 | -3.39 | 0 | NRF |
| CV_1949 |  | hypothetical | 27.34 | 2.16 | -3.66 | 3.01E-005 | NRF |
| CV_0058 |  | hypothetical protein | 211.37 | 20.93 | -3.34 | 1.09E-006 | NRF |
| CV_0497 |  | hypothetical protein | 232.28 | 28.19 | -3.04 | 0 | NRF |
| CV_1001 |  | hypothetical protein | 372.43 | 42.47 | -3.13 | 4.14E-005 | NRF |
| CV_0015 |  | hypothetical protein | 20.88 | 2.11 | -3.31 | 0 | NRF |
| CV_0242 |  | hypothetical protein | 212.65 | 14.79 | -3.85 | 7.20E-005 | NRF |
| CV_0407 |  | hypothetical protein | 88.68 | 10.54 | -3.07 | 0 | NRF |
| CV_0454 |  | hypothetical protein | 123.74 | 7.85 | -3.98 | 4.11E-005 | NRF |
| CV_0565 |  | hypothetical protein | 100.65 | 2.43 | -5.37 | 0 | NRF |
| CV_0567 |  | hypothetical protein | 41.49 | 0.71 | -5.87 | 8.15E-005 | NRF |
| CV_0570 |  | hypothetical protein | 41.49 | 0.71 | -5.87 | 8.15E-005 | NRF |
| CV_0583 |  | hypothetical protein | 169.08 | 19.69 | -3.1 | 1.20E-005 | NRF |
| CV_0584 |  | hypothetical protein | 169.08 | 19.69 | -3.1 | 1.20E-005 | NRF |
| CV_0602 |  | hypothetical protein | 511.35 | 2.34 | -7.77 | 2.38E-007 | NRF |
| CV_0638 |  | hypothetical protein | 12.9 | 0.77 | -4.07 | 0.01 | NRF |
| CV_0840 |  | hypothetical protein | 26.59 | 2.09 | -3.67 | 0 | NRF |
| CV_0860 |  | hypothetical protein | 9.01 | 0.31 | -4.88 | 0 | NRF |
| CV_0904 |  | hypothetical protein | 38.97 | 4.46 | -3.13 | 0.01 | NRF |
| CV_1015 |  | hypothetical protein | 18.23 | 1.14 | -4 | 0 | NRF |
| CV_1050 |  | hypothetical protein | 69.59 | 3.81 | -4.19 | 0.01 | NRF |
| CV_1052 |  | hypothetical protein | 99.15 | 3.59 | -4.79 | 1.03E-007 | NRF |
| CV_1053 |  | hypothetical protein | 67.16 | 6.32 | -3.41 | 3.01E-005 | NRF |
| CV_1082 |  | hypothetical protein | 177.16 | 6.67 | -4.73 | 3.15E-005 | NRF |
| CV_1172 |  | hypothetical protein | 682.99 | 51.04 | -3.74 | 9.79E-005 | NRF |
| CV_1207 |  | hypothetical protein | 833.13 | 100.64 | -3.05 | 0 | NRF |
| CV_1209 |  | hypothetical protein | 1166.09 | 43.95 | -4.73 | 1.40E-009 | NRF |
| CV_1304 |  | hypothetical protein | 36.09 | 2 | -4.18 | 3.71E-007 | NRF |
| CV_1311 |  | hypothetical protein | 46.41 | 1.6 | -4.86 | 0 | NRF |
| CV_1356 |  | hypothetical protein | 393372 | 13826.4 | -4.83 | 2.89E-010 | NRF |
| CV_1444 |  | hypothetical protein | 14.81 | 0.94 | -3.98 | 0.01 | NRF |
| CV_1692 |  | hypothetical protein | 53.53 | 2.44 | -4.46 | 0 | NRF |
| CV_1728 |  | hypothetical protein | 40.69 | 2.28 | -4.16 | 2.62E-006 | NRF |
| CV_1783 |  | hypothetical protein | 98.18 | 10.07 | -3.28 | 0 | NRF |
| CV_1784 |  | hypothetical protein | 397.09 | 37.37 | -3.41 | 7.41E-006 | NRF |
| CV_1792 |  | hypothetical protein | 116.94 | 12 | -3.28 | 0 | NRF |
| CV_1831 |  | hypothetical protein | 181.09 | 10.4 | -4.12 | 0.01 | NRF |
| CV_1890 |  | hypothetical protein | 137.3 | 8.9 | -3.95 | 0 | NRF |
| CV_1899 |  | hypothetical protein | 32.64 | 0.62 | -5.71 | 0 | NRF |
| CV_1997 |  | hypothetical protein | 1491.86 | 104.02 | -3.84 | 1.59E-006 | NRF |
| CV_2029 |  | hypothetical protein | 43.85 | 2.91 | -3.91 | 5.06E-005 | NRF |
| CV_2077 |  | hypothetical protein | 44.28 | 3.64 | -3.61 | 0 | NRF |
| CV_2125 |  | hypothetical protein | 33.87 | 3.51 | -3.27 | 0 | NRF |
| CV_2126 |  | hypothetical protein | 33.87 | 3.51 | -3.27 | 0 | NRF |
| CV_2212 |  | hypothetical protein | 561.06 | 41.05 | -3.77 | 0 | NRF |
| CV_2411 |  | hypothetical protein | 48.38 | 4.49 | -3.43 | 0 | NRF |
| CV_2414 |  | hypothetical protein | 12.87 | 0.82 | -3.98 | 0.01 | NRF |
| CV_2435 |  | hypothetical protein | 56.1 | 1.93 | -4.86 | 0 | NRF |
| CV_2561 |  | hypothetical protein | 27.96 | 2.06 | -3.76 | 0 | NRF |
| CV_2716 |  | hypothetical protein | 52.45 | 5.47 | -3.26 | 4.07E-005 | NRF |
| CV_2820 |  | hypothetical protein | 491.54 | 31.21 | -3.98 | 4.13E-005 | NRF |
| CV_2870 |  | hypothetical protein | 14.84 | 0.71 | -4.38 | 0.01 | NRF |
| CV_2906 |  | hypothetical protein | 19.95 | 0.78 | -4.68 | 0 | NRF |
| CV_2949 |  | hypothetical protein | 88.28 | 10.95 | -3.01 | 6.03E-005 | NRF |
| CV_2950 |  | hypothetical protein | 88.28 | 10.95 | -3.01 | 6.03E-005 | NRF |
| CV_2975 |  | hypothetical protein | 53.86 | 2.4 | -4.49 | 0 | NRF |
| CV_3214 |  | hypothetical protein | 56.65 | 6.21 | -3.19 | 0 | NRF |
| CV_3400 |  | hypothetical protein | 643.95 | 64.05 | -3.33 | 4.07E-005 | NRF |
| CV_3599 |  | hypothetical protein | 26.7 | 0.71 | -5.23 | 0 | NRF |
| CV_3830 |  | hypothetical protein | 3313.59 | 70.85 | -5.55 | 1.35E-006 | NRF |
| CV_3835 |  | hypothetical protein | 133.99 | 8.54 | -3.97 | 1.92E-007 | NRF |
| CV_3872 |  | hypothetical protein | 266.17 | 26.78 | -3.31 | 8.05E-007 | NRF |
| CV_3873 |  | hypothetical protein | 266.17 | 26.78 | -3.31 | 8.05E-007 | NRF |
| CV_3968 |  | hypothetical protein | 21.1 | 0.95 | -4.47 | 0 | NRF |
| CV_4036 |  | hypothetical protein | 78.18 | 1.11 | -6.14 | 6.52E-005 | NRF |
| CV_4071 |  | hypothetical protein | 446.83 | 44.95 | -3.31 | 0 | NRF |
| CV_4093 |  | hypothetical protein | 52.29 | 5.14 | -3.35 | 3.77E-005 | NRF |
| CV_4106 |  | hypothetical protein | 22.62 | 0.52 | -5.45 | 0 | NRF |
| CV_4312 |  | hypothetical protein | 2627.75 | 216.24 | -3.6 | 4.14E-006 | NRF |
| CV_4334 |  | hypothetical protein | 79.58 | 6.26 | -3.67 | 0 | NRF |
| CV_3867 |  | hypothetical protein | 51.31 | 3.26 | -3.98 | 0.01 | NRF |
| CV_2517 | *dedD* | lipoprotein | 31.12 | 1.44 | -4.43 | 0 | NRF |
| CV_1367 | *phbF* | PhbF protein | 119.94 | 13.35 | -3.17 | 0 | NRF |
| CV_0415 |  | probable bacteriophage tail fibre protein | 78.07 | 1.87 | -5.39 | 7.19E-006 | NRF |
| CV_2935 |  | probable chitinase A | 30.27 | 2.15 | -3.82 | 8.06E-005 | NRF |
| CV_4276 |  | probable glutamate-cysteine ligase | 32.42 | 2.21 | -3.87 | 6.41E-005 | NRF |
| CV_3897 |  | probable hemin degrading factor | 291.51 | 24.96 | -3.55 | 2.81E-006 | NRF |
| CV_3458 |  | probable phosphopyruvate hydratase | 138.67 | 10.78 | -3.68 | 7.12E-006 | NRF |
| CV_0181 |  | probable response regulator | 11.81 | 0.59 | -4.32 | 0 | NRF |
| CV_1149 |  | probable RNA methyltransferase | 46.23 | 4.4 | -3.39 | 0 | NRF |
| CV_3512 |  | probable signal peptide protein | 708.06 | 47.46 | -3.9 | 9.49E-006 | NRF |
| CV_0210 | *ohrR* | probable transcriptional regulator, OhrR | 110.72 | 7.42 | -3.9 | 0 | NRF |
| CV_3504 |  | probable two-component response regulator | 34.54 | 2.41 | -3.84 | 0 | NRF |
| CV_0956 | *rna* | RNase | 54.73 | 6 | -3.19 | 0 | NRF |
| CV_2601 | *ssaM* | Secretion system apparatus | 19.3 | 0.43 | -5.48 | 0 | NRF |
| CV_2577 | *sseC* | secretion system effector | 14.97 | 1.24 | -3.59 | 0 | NRF |
| CV_2574 | *sseE* | secretion system effector SseE | 52.45 | 3.51 | -3.9 | 0.01 | NRF |
| CV_0832 | *pilN* | type 4 fimbrial biogenesis protein PilN | 85.03 | 6.41 | -3.73 | 0 | NRF |
| CV_3693 | *cheR3* | chemotaxis protein methyltransferase | 41.45 | 3.66 | -3.5 | 0 | N |
| CV_2507 | *cheR1* | probable chemotaxis protein methyltransferase | 27.81 | 2.73 | -3.35 | 0 | N |
| CV_2506 | *cheB1* | protein-glutamate methylesterase | 27.81 | 2.73 | -3.35 | 0 | N |
| CV_1799 | *ate1* | arginyltransferase | 487.61 | 58.07 | -3.07 | 7.01E-006 | O |
| CV_2815 | *bcp* | bacterioferritin comigratory protein | 310.02 | 21.99 | -3.82 | 5.45E-006 | O |
| CV_3460 |  | conserved hypothetical protein | 132.3 | 15.2 | -3.12 | 0.01 | O |
| CV_3057 |  | conserved hypothetical protein | 26.5 | 1.84 | -3.85 | 0.01 | O |
| CV_0606 | *ctaA* | cytochrome aa3 oxidase assembly protein | 153.01 | 12.07 | -3.66 | 5.27E-009 | O |
| CV_3582 | *slyD* | fkbp-type peptidyl-prolyl cis-trans isomerase | 121.44 | 14.35 | -3.08 | 0 | O |
| CV_1126 | *grxC* | glutaredoxin 3 | 1307.95 | 35.59 | -5.2 | 1.55E-007 | O |
| CV_1164 | *gst1* | glutathione S-transferase family protein | 26.89 | 1.91 | -3.82 | 0.01 | O |
| CV_1798 | *aat* | leucyltransferase | 487.61 | 58.07 | -3.07 | 7.01E-006 | O |
| CV_3965 |  | probable ClpA/B-type chaperone | 15.6 | 1.65 | -3.24 | 6.90E-005 | O |
| CV_1987 |  | probable metallopeptidase | 39.05 | 4.88 | -3 | 0 | O |
| CV_4305 |  | probable metallopeptidase | 14.99 | 1.85 | -3.02 | 0 | O |
| CV_2934 |  | probable peptidyl-prolyl cis-trans isomerase | 45.29 | 4.55 | -3.32 | 0 | O |
| CV_1968 |  | probable protease | 12.65 | 0.54 | -4.54 | 0 | O |
| CV_0234 | *pcm2* | protein-L-isoaspartate(D-aspartate) O-methyltransferase | 59.34 | 5.23 | -3.5 | 0 | O |
| CV_2917 | *glnD* | protein-PII uridylyltransferase | 8.41 | 1.01 | -3.05 | 0 | O |
| CV_2095 | *glnE* | glutamate-ammonia-ligase adenylyltransferase | 10.43 | 1.29 | -3.02 | 0 | O |
| CV_2104 | *cysC* | adenylyl-sulfate kinase | 14.27 | 0.91 | -3.96 | 0.01 | P |
| CV_0233 |  | conserved hypothetical protein | 88.45 | 6.27 | -3.82 | 0.01 | P |
| CV_1457 |  | conserved hypothetical protein | 59.85 | 4.29 | -3.8 | 5.59E-007 | P |
| CV_3217 | *copF* | Cu-ATPase | 6.73 | 0.81 | -3.05 | 0.01 | P |
| CV_2230 | *fepA* | enterobactin-iron outermembrane receptor protein | 60.91 | 3.45 | -4.14 | 7.10E-007 | P |
| CV_2231 | *fes* | enterochelin esterase | 289.4 | 26.11 | -3.47 | 4.11E-006 | P |
| CV_1797 | *fur* | ferric uptake regulation protein | 487.61 | 58.07 | -3.07 | 7.01E-006 | P |
| CV_2239 | *fepB* | ferrienterobactin-binding periplasmic protein precursor | 30.27 | 2.09 | -3.86 | 0 | P |
| CV_3326 | *kefB* | glutathione-regulated potassium-efflux system protein | 26.64 | 2.71 | -3.3 | 9.93E-005 | P |
| CV_0934 | *pitA* | low-affinity inorganic phosphate transporter 1 | 9 | 0.64 | -3.82 | 0.01 | P |
| CV_3727 | *modB* | molybdate transport system permease protein | 316.33 | 21.83 | -3.86 | 8.88E-006 | P |
| CV_3726 | *modA* | molybdate-binding periplasmic protein precursor | 316.33 | 21.83 | -3.86 | 8.88E-006 | P |
| CV_1261 | *rpiA* | phosphate transport system regulatory protein | 112.88 | 5.23 | -4.43 | 5.58E-006 | P |
| CV_1109 |  | probable ATP-sensitive inward rectifier potassium channel related transmembrane protein | 22.14 | 1.16 | -4.25 | 0.01 | P |
| CV_3677 |  | probable cation efflux system | 26.3 | 2.26 | -3.54 | 2.00E-006 | P |
| CV_2042 |  | probable cation transport P-type ATPase | 169.34 | 1.8 | -6.56 | 6.80E-012 | P |
| CV_4147 |  | probable cation-efflux system transmembrane protein | 48.6 | 3.25 | -3.9 | 9.43E-006 | P |
| CV_1414 |  | probable copper homeostasis protein | 96.64 | 7.37 | -3.71 | 6.20E-006 | P |
| CV_3562 |  | probable ion transporter | 18.87 | 1.2 | -3.98 | 0.01 | P |
| CV_3907 |  | probable nitrite extrusion protein | 156.81 | 7.39 | -4.41 | 5.66E-008 | P |
| CV_1722 |  | probable sulfate permease family protein | 12.23 | 0.55 | -4.49 | 0 | P |
| CV_1982 |  | probable tonB dependent receptor | 39.48 | 1.66 | -4.57 | 5.95E-008 | P |
| CV_3896 |  | probable TonB-dependent receptor | 173.75 | 9.97 | -4.12 | 5.24E-008 | P |
| CV_1970 |  | probable TonB-dependent receptor protein | 20.23 | 1.35 | -3.9 | 9.43E-006 | P |
| CV_3619 |  | probable voltage-gated chloride channel family protein | 27.32 | 0.69 | -5.32 | 0 | P |
| CV_2234 | *frdD* | enterobactin-iron transport system ATP-binding protein | 127.92 | 15.39 | -3.05 | 5.69E-005 | P |
| CV_2234 | *fepC* | enterobactin-iron transport system ATP-binding protein | 131.19 | 12.09 | -3.44 | 5.34E-010 | P |
| CV_2236 | *fepD* | enterobactin-iron transport system permease protein | 131.19 | 12.09 | -3.44 | 5.34E-010 | P |
| CV_2235 | *fepG* | enterobactin-iron transport system permease protein | 131.19 | 12.09 | -3.44 | 5.34E-010 | P |
| CV_2236 | *ftsI* | enterobactin-iron transport system permease protein | 157.11 | 0.52 | -8.25 | 3.40E-008 | P |
| CV_3899 | *fecE* | ferric citrate transport system ATP-binding protein | 125.91 | 6.73 | -4.22 | 1.05E-008 | P |
| CV_3898 |  | probable permease of ABC transporter | 125.91 | 6.73 | -4.22 | 1.05E-008 | P |
| CV_1816 |  | conserved hypothetical protein | 31.22 | 3.51 | -3.15 | 0 | P |
| CV_1484 | *entE* | 2,3-dihydroxybenzoate-AMP ligase | 480.25 | 29.46 | -4.03 | 8.44E-014 | Q |
| CV_3412 | *fabF* | 3-oxoacyl-[acyl-carrier-protein] synthase II | 28.71 | 3.08 | -3.22 | 0 | Q |
| CV_2241 | *acrB* | acriflavin resistance protein B | 13.9 | 0.27 | -5.67 | 0 | Q |
| CV_0434 | *acrD* | acriflavin resistance protein D | 55.32 | 4.15 | -3.74 | 1.83E-008 | Q |
| CV_2455 | *vmrA* | cation (Na+-coupled) multidrug resistance efflux pump | 19.18 | 0.7 | -4.78 | 0 | Q |
| CV_0466 |  | conserved hypothetical protein | 235.52 | 10.63 | -4.47 | 6.56E-013 | Q |
| CV_1486 | *entF* | enterobactin synthetase component F | 8.17 | 0.53 | -3.96 | 0 | Q |
| CV_0970 | *hmgA* | homogentisate 1,2-dioxygenase | 93.27 | 9.44 | -3.3 | 2.47E-005 | Q |
| CV_1976 | *nolG* | NolG efflux transporter | 12.97 | 1.37 | -3.24 | 6.91E-005 | Q |
| CV_0437 |  | probable ABC transporter ATP-binding protein | 27.25 | 1.14 | -4.58 | 0 | Q |
| CV_2459 |  | probable ABC transporter system, ATP-binding protein | 8.38 | 0.54 | -3.96 | 0.01 | Q |
| CV_4070 |  | probable ABC transporter, ATP-binding protein | 15.59 | 1.93 | -3.02 | 0 | Q |
| CV_0307 |  | probable ABC transporter, ATP-binding/permease fusion | 10.8 | 0.81 | -3.73 | 0 | Q |
| CV_0516 |  | probable calcium binding hemolysin | 7.43 | 0.71 | -3.39 | 3.36E-005 | Q |
| CV_0068 |  | probable colicin V secretion atp-binding protein | 9.71 | 1.21 | -3 | 0 | Q |
| CV_2656 |  | probable cytochrome P450 hydroxylase | 96.94 | 8.63 | -3.49 | 7.18E-006 | Q |
| CV_0905 |  | probable glutathione S-transferase family protein | 38.97 | 4.46 | -3.13 | 0.01 | Q |
| CV_2788 |  | probable isomerase | 133.71 | 11.41 | -3.55 | 4.29E-005 | Q |
| CV_2705 |  | probable membrane protein | 51.24 | 4.17 | -3.62 | 6.15E-008 | Q |
| CV_0435 |  | probable multidrug efflux membrane permease | 43.74 | 1.62 | -4.75 | 2.89E-005 | Q |
| CV_2240 |  | probable multidrug efflux protein | 29.75 | 1.67 | -4.16 | 0 | Q |
| CV_0311 |  | probable RTX (repeat in structural toxin) | 123.92 | 14.64 | -3.08 | 0 | Q |
| CV_2233 | *cbsF/entF* | synthetase CbsF | 7.93 | 0.57 | -3.79 | 9.49E-006 | Q |
| CV_1482 | *entA* | 2,3-dihydro-2,3-dihydroxybenzoate dehydrogenase | 207.29 | 7.1 | -4.87 | 1.09E-008 | Q |
| CV_1482 | *entB* | 2,3-dihydro-2,3-dihydroxybenzoate dehydrogenase | 254.73 | 22.44 | -3.5 | 4.41E-006 | Q |
| CV_4376 | *bioC* | biotin synthesis protein | 18.77 | 1.13 | -4.05 | 0.01 | Q |
| CV_0334 |  | conserved hypothetical protein | 94.4 | 8.56 | -3.46 | 6.13E-007 | Q |
| CV_1741 |  | conserved hypothetical protein | 42.27 | 2.01 | -4.4 | 0 | Q |
| CV_2028 |  | Conserved hypothetical protein | 43.85 | 2.91 | -3.91 | 5.06E-005 | Q |
| CV_1221 |  | hypothetical protein | 43.03 | 4.7 | -3.19 | 0 | Q |
| CV_4378 |  | probable phosphatidylethanolamine N-methyltransferase | 16.18 | 0.61 | -4.74 | 1.65E-005 | Q |
| CV_1942 |  | probable peptidase | 13.42 | 0.9 | -3.9 | 0 | Q |
| CV_1331 | *pcaD* | 3-oxoadipate enol-lactonase | 48.58 | 4.09 | -3.57 | 0 | R |
| CV_3635 | *dcuA* | anaerobic C4-dicarboxylate membrane transporter protein | 40.09 | 2.16 | -4.21 | 1.49E-005 | R |
| CV_4377 | *bioH* | BioH protein | 18.77 | 1.13 | -4.05 | 0.01 | R |
| CV_2675 | *bscC* | cellulose synthase, subunit C | 9.3 | 0.22 | -5.39 | 0 | R |
| CV_2370 | *cinA* | cinA-related protein | 102.37 | 9.17 | -3.48 | 2.66E-008 | R |
| CV_1561 | *cobW* | cobalamin synthesis protein | 9.36 | 0.6 | -3.97 | 0.01 | R |
| CV_4382 | *comF* | competence protein F | 26.97 | 1.71 | -3.98 | 0.01 | R |
| CV_0273 |  | conserved hypothetical protein | 23.02 | 1.63 | -3.82 | 0 | R |
| CV_0608 |  | conserved hypothetical protein | 153.01 | 12.07 | -3.66 | 5.27E-009 | R |
| CV_0912 |  | conserved hypothetical protein | 88.57 | 7.91 | -3.49 | 6.82E-005 | R |
| CV_0924 |  | conserved hypothetical protein | 75.64 | 8.55 | -3.15 | 7.14E-005 | R |
| CV_1362 |  | conserved hypothetical protein | 19.91 | 1.92 | -3.37 | 0 | R |
| CV_1693 |  | conserved hypothetical protein | 53.53 | 2.44 | -4.46 | 0 | R |
| CV_1755 |  | conserved hypothetical protein | 22.22 | 2.32 | -3.26 | 0 | R |
| CV_2352 |  | conserved hypothetical protein | 23.03 | 2.69 | -3.1 | 0 | R |
| CV_2356 |  | conserved hypothetical protein | 30.33 | 1.83 | -4.05 | 0.01 | R |
| CV_2373 |  | conserved hypothetical protein | 77.55 | 3.69 | -4.39 | 6.61E-006 | R |
| CV_2500 |  | conserved hypothetical protein | 54.43 | 1.96 | -4.79 | 0 | R |
| CV_2670 |  | conserved hypothetical protein | 16.26 | 1.4 | -3.54 | 0 | R |
| CV_2696 |  | conserved hypothetical protein | 45.86 | 2.71 | -4.08 | 3.97E-007 | R |
| CV_2715 |  | conserved hypothetical protein | 23.01 | 2.62 | -3.13 | 0 | R |
| CV_2925 |  | conserved hypothetical protein | 28.02 | 2.18 | -3.68 | 0 | R |
| CV_3155 |  | conserved hypothetical protein | 55.92 | 5.39 | -3.37 | 0 | R |
| CV_3541 |  | conserved hypothetical protein | 79.43 | 9.57 | -3.05 | 1.09E-006 | R |
| CV_3607 |  | conserved hypothetical protein | 34.84 | 3.18 | -3.45 | 0 | R |
| CV_4061 |  | conserved hypothetical protein | 92.22 | 9.61 | -3.26 | 9.85E-006 | R |
| CV_2653 | *csaA* | CsaA protein | 77.94 | 5.53 | -3.82 | 0.01 | R |
| CV_4385 | *engB* | GTP-binding protein | 58.52 | 3.81 | -3.94 | 0 | R |
| CV_2067 | *era* | GTP-binding protein | 123.72 | 10.35 | -3.58 | 9.49E-009 | R |
| CV_1684 | *hcnA* | hydrogen cyanide synthase HcnA | 600.99 | 4.65 | -7.01 | 0 | R |
| CV_1683 | *hcnB* | hydrogen cyanide synthase HcnB | 600.99 | 4.65 | -7.01 | 0 | R |
| CV_2068 |  | hypothetical protein | 123.72 | 10.35 | -3.58 | 9.49E-009 | R |
| CV_0776 | *thrB* | ketohexokinase | 17.93 | 1.08 | -4.05 | 0.01 | R |
| CV_2964 | *lolC* | lipoprotein releasing system trasmembrane protein | 46.91 | 1.54 | -4.93 | 5.34E-006 | R |
| CV_4153 | *corC* | magnesium and cobalt efflux protein corC | 73.47 | 4.92 | -3.9 | 9.43E-006 | R |
| CV_2547 | *pqiB* | paraquat-inducible protein B | 77.79 | 6.98 | -3.48 | 7.20E-006 | R |
| CV_2458 |  | probable ABC transport system, permease protein | 8.38 | 0.54 | -3.96 | 0.01 | R |
| CV_2704 |  | probable ABC transporter, ATP-binding protein | 51.24 | 4.17 | -3.62 | 6.15E-008 | R |
| CV_0335 |  | probable alanyl-tRNA synthetase related protein | 94.4 | 8.56 | -3.46 | 6.13E-007 | R |
| CV_2944 |  | probable amidotransferase | 63.92 | 2.75 | -4.54 | 6.36E-005 | R |
| CV_0056 |  | probable aminopeptidase | 12.69 | 1.53 | -3.05 | 0.01 | R |
| CV_2963 |  | probable ATP-binding component, ABC transporter | 46.91 | 1.54 | -4.93 | 5.34E-006 | R |
| CV_0416 |  | probable bacteriophage regulatory protein | 78.07 | 1.87 | -5.39 | 7.19E-006 | R |
| CV_0739 |  | probable esterase | 74.09 | 8.93 | -3.05 | 4.58E-006 | R |
| CV_1591 |  | probable haloacid dehalogenase-like hydrolase | 68.38 | 3.93 | -4.12 | 0 | R |
| CV_3342 |  | probable hemolysin III | 274 | 26.88 | -3.35 | 1.39E-005 | R |
| CV_3718 |  | probable hydrolase protein | 25.27 | 1.45 | -4.12 | 0.01 | R |
| CV_2322 |  | probable inorganic polyphosphate/ATP-NAD kinase | 55.11 | 3.56 | -3.95 | 4.59E-005 | R |
| CV_3685 |  | probable MoxR like protein | 41.81 | 3.35 | -3.64 | 7.59E-005 | R |
| CV_0530 |  | probable NADPH:quinone oxidoreductase | 52.51 | 5.5 | -3.25 | 0 | R |
| CV_3691 |  | probable oxygen-binding protein | 63.23 | 4.24 | -3.9 | 0.01 | R |
| CV_1817 |  | probable phosphoglycolate phosphatase | 31.22 | 3.51 | -3.15 | 0 | R |
| CV_2380 |  | probable sodium-dependent transporter | 11.26 | 1.29 | -3.12 | 0.01 | R |
| CV_2697 |  | probable tldD protein family | 45.86 | 2.71 | -4.08 | 3.97E-007 | R |
| CV_1537 |  | probable transcriptional accessory protein | 40.26 | 1.9 | -4.41 | 1.42E-007 | R |
| CV_0438 |  | probable transmenbrane protein | 27.25 | 1.14 | -4.58 | 0 | R |
| CV_2714 |  | probable triacylglycerol lipase | 26.32 | 2.19 | -3.59 | 0 | R |
| CV_0544 | *NifR3* | Regulatory gene required to sense and relay the nitrogen status | 37.16 | 3.73 | -3.32 | 0 | R |
| CV_2620 | *spaT* | surface presentation of antigens; secretory proteins | 116.65 | 3.16 | -5.21 | 6.23E-005 | R |
| CV_4403 | *thdF* | thiophene and furan oxidation protein ThdF | 40.36 | 3.47 | -3.54 | 1.55E-005 | R |
| CV_4362 | *pqqL* | zinc protease | 11.67 | 0.94 | -3.64 | 0 | R |
| CV_3370 | *dcuB* | anaerobic C4-dicarboxylate membrane transporter protein | 17.67 | 2.13 | -3.05 | 0 | R |
| CV_1314 |  | probable transcriptional regulator, AraC family | 19.06 | 1.09 | -4.12 | 0.01 | R |
| CV_2101 |  | probable transcriptional regulator, AraC family | 50.15 | 0.93 | -5.75 | 0 | R |
| CV_2076 |  | conserved hypothetical protein | 82.99 | 8.47 | -3.29 | 0 | R |
| CV_4030 |  | probable sugar-phosphate nucleotide transferase | 21.53 | 0.95 | -4.51 | 0 | R |
| CV_1368 |  | conserved hypothetical protein | 12.03 | 0.73 | -4.05 | 0.01 | R |
| CV_3088 | *argR* | transcriptional regulator (AraC-type DNA-binding domain-containing proteins) | 15.04 | 1.01 | -3.9 | 0.01 | R |
| CV_0155 |  | conserved hypothetical protein | 67.66 | 3.71 | -4.19 | 0.01 | S |
| CV_0184 |  | conserved hypothetical protein | 26.93 | 1.37 | -4.3 | 0 | S |
| CV_0482 |  | conserved hypothetical protein | 7.05 | 0.27 | -4.68 | 0 | S |
| CV_0498 |  | conserved hypothetical protein | 232.28 | 28.19 | -3.04 | 0 | S |
| CV_0763 |  | conserved hypothetical protein | 48.31 | 2.91 | -4.05 | 2.99E-005 | S |
| CV_0913 |  | conserved hypothetical protein | 48.05 | 3.41 | -3.82 | 8.06E-005 | S |
| CV_1069 |  | conserved hypothetical protein | 131.48 | 6.28 | -4.39 | 9.66E-008 | S |
| CV_1144 |  | conserved hypothetical protein | 53.86 | 4.81 | -3.49 | 0 | S |
| CV_1250 |  | conserved hypothetical protein | 24.34 | 2.79 | -3.12 | 0.01 | S |
| CV_1252 |  | conserved hypothetical protein | 25.2 | 1.86 | -3.76 | 0 | S |
| CV_1756 |  | conserved hypothetical protein | 22.22 | 2.32 | -3.26 | 0 | S |
| CV_1952 |  | conserved hypothetical protein | 24.37 | 1.28 | -4.25 | 0.01 | S |
| CV_2052 |  | Conserved hypothetical protein | 289.35 | 14.24 | -4.34 | 1.00E-006 | S |
| CV_2053 |  | conserved hypothetical protein | 129.5 | 2.43 | -5.74 | 1.08E-008 | S |
| CV_2727 |  | conserved hypothetical protein | 453.36 | 17.49 | -4.7 | 1.63E-007 | S |
| CV_3029 |  | conserved hypothetical protein | 113.73 | 13.68 | -3.06 | 3.57E-009 | S |
| CV_3191 |  | conserved hypothetical protein | 8.98 | 0.6 | -3.9 | 0.01 | S |
| CV_3280 |  | conserved hypothetical protein | 61.03 | 4.07 | -3.91 | 9.02E-005 | S |
| CV_3345 |  | conserved hypothetical protein | 368.3 | 5.77 | -6 | 1.91E-011 | S |
| CV_3550 |  | conserved hypothetical protein | 24.09 | 1.38 | -4.12 | 0.01 | S |
| CV_3684 |  | conserved hypothetical protein | 41.81 | 3.35 | -3.64 | 7.59E-005 | S |
| CV_3961 |  | conserved hypothetical protein | 35.43 | 3.05 | -3.54 | 0 | S |
| CV_3967 |  | conserved hypothetical protein | 21.1 | 0.95 | -4.47 | 0 | S |
| CV_4352 |  | conserved hypothetical protein | 157.11 | 0.52 | -8.25 | 3.40E-008 | S |
| CV_0111 |  | conserved hypothetical protein | 103.33 | 8.9 | -3.54 | 3.20E-005 | S |
| CV_3147 |  | conserved hypothetical protein | 19.31 | 1.47 | -3.71 | 0.01 | S |
| CV_0569 |  | hypothetical protein | 41.49 | 0.71 | -5.87 | 8.15E-005 | S |
| CV_0582 |  | hypothetical protein | 169.08 | 19.69 | -3.1 | 1.20E-005 | S |
| CV_0604 |  | hypothetical protein | 113.34 | 7.22 | -3.97 | 1.89E-006 | S |
| CV_2041 | *fixS* | nitrogen fixation protein | 169.34 | 1.8 | -6.56 | 6.80E-012 | S |
| CV_2548 | *pqiA* | paraquat-inducible protein A | 77.79 | 6.98 | -3.48 | 7.20E-006 | S |
| CV_3871 |  | probable flagellar biosynthesis-related protein | 266.17 | 26.78 | -3.31 | 8.05E-007 | S |
| CV_2549 |  | probable transmembrane protein | 77.79 | 6.98 | -3.48 | 7.20E-006 | S |
| CV_1720 |  | probable UDP-galactose-lipid carrier transferase | 226.52 | 12.3 | -4.2 | 1.95E-007 | S |
| CV_0016 |  | probable vgr related protein | 20.88 | 2.11 | -3.31 | 0 | S |
| CV_1824 |  | conserved hypothetical protein | 70.56 | 5.13 | -3.78 | 6.52E-006 | S |
| CV_1567 | *cbiC* | precorrin-8X methylmutase | 59.37 | 2.77 | -4.42 | 5.46E-006 | S |
| CV_0396 | *aer* | aerotaxis receptor | 17.92 | 1.31 | -3.77 | 0 | T |
| CV_4067 | *apaH* | bis(5'-nucleosyl)-tetraphosphatase | 25.19 | 1.26 | -4.32 | 0 | T |
| CV_0841 |  | conserved hypothetical protein | 8.2 | 0.99 | -3.05 | 0.01 | T |
| CV_0137 |  | conserved hypothetical protein | 24.09 | 2.77 | -3.12 | 0.01 | T |
| CV_0240 |  | conserved hypothetical protein | 15.05 | 0.86 | -4.12 | 0.01 | T |
| CV_0542 |  | conserved hypothetical protein | 8.98 | 0.94 | -3.25 | 0 | T |
| CV_0657 |  | conserved hypothetical protein | 31.16 | 2.4 | -3.7 | 0 | T |
| CV_1034 |  | conserved hypothetical protein | 11.49 | 0.21 | -5.75 | 0 | T |
| CV_1143 |  | conserved hypothetical protein | 19.4 | 2.06 | -3.23 | 0 | T |
| CV_2376 |  | conserved hypothetical protein | 296.15 | 35.38 | -3.07 | 7.87E-005 | T |
| CV_2398 |  | conserved hypothetical protein | 88.33 | 7.1 | -3.64 | 9.21E-006 | T |
| CV_2953 |  | conserved hypothetical protein | 14.38 | 1.21 | -3.57 | 2.72E-005 | T |
| CV_4300 |  | conserved hypothetical protein | 611.37 | 8.7 | -6.13 | 1.17E-009 | T |
| CV_4379 |  | conserved hypothetical protein | 16.18 | 0.61 | -4.74 | 1.65E-005 | T |
| CV_3148 |  | conserved hypothetical protein | 19.31 | 1.47 | -3.71 | 0.01 | T |
| CV_1110 |  | hypothetical protein | 8.76 | 1.01 | -3.12 | 0.01 | T |
| CV_0563 | *phoR* | phosphate regulon sensor protein phoR | 17.28 | 1.45 | -3.58 | 0 | T |
| CV_3082 |  | probable GGDEF family regulatory protein | 14.65 | 0.98 | -3.9 | 0.01 | T |
| CV_2498 | *hik2* | probable hybrid sensory kinase | 20.88 | 0.86 | -4.61 | 0 | T |
| CV_2213 |  | probable sensor histidine kinase/response regulator | 19.78 | 0.8 | -4.63 | 4.55E-005 | T |
| CV_1771 |  | probable sensor kinase protein | 11.44 | 0.65 | -4.14 | 0.01 | T |
| CV_2643 |  | probable sensor/response regulator hybrid | 10.67 | 0.78 | -3.78 | 1.74E-005 | T |
| CV_1718 |  | probable sensor/response regulator hybrid | 8.95 | 0.54 | -4.05 | 0.01 | T |
| CV_3503 |  | probable sensor/response regulator hybrid | 34.54 | 2.41 | -3.84 | 0 | T |
| CV_1621 |  | probable sensor/response regulatory hybrid protein | 11.45 | 1.43 | -3 | 0 | T |
| CV_3259 |  | probable sensory transduction histidine kinase | 9.89 | 0.7 | -3.81 | 0 | T |
| CV_1664 |  | probable transcriptional regulator protein | 20.34 | 0.51 | -5.32 | 0 | T |
| CV_0688 |  | probable transcriptional regulatory protein | 12.89 | 0.71 | -4.19 | 0.01 | T |
| CV_2497 |  | probable two component system, transcriptional regulatory protein | 20.88 | 0.86 | -4.61 | 0 | T |
| CV_3138 |  | probable two-component response regulator | 131.04 | 8.17 | -4 | 4.02E-007 | T |
| CV_1951 |  | probable two-component sensor | 28.57 | 0.54 | -5.73 | 0 | T |
| CV_2324 |  | probable two-component sensor | 63.35 | 5.66 | -3.48 | 8.76E-006 | T |
| CV_0760 |  | probable two-component sensor protein | 16.72 | 2.08 | -3 | 0 | T |
| CV_3844 |  | probable two-component sensor protein | 13.61 | 1.49 | -3.19 | 0 | T |
| CV_1189 |  | probable two-component system | 38.93 | 2.93 | -3.73 | 0 | T |
| CV_1665 |  | probable two-component system sensor kinase | 20.34 | 0.51 | -5.32 | 0 | T |
| CV_3089 |  | probable two-component system | 24.18 | 1.53 | -3.98 | 0 | T |
| CV_1770 |  | probable two-component transcriptional regulator | 11.44 | 0.65 | -4.14 | 0.01 | T |
| CV_3340 | *hydH* | two-component sensor histidine kinase protein | 104.45 | 9.52 | -3.46 | 9.93E-009 | T |
| CV_3438 |  | probable methyl-accepting chemotaxis protein | 15.92 | 0.58 | -4.78 | 2.67E-005 | T |
| CV_1454 |  | probable methyl-accepting chemotaxis protein | 18.16 | 2.12 | -3.1 | 0 | T |
| CV_1374 |  | conserved hypothetical protein | 16.83 | 1.03 | -4.03 | 3.32E-005 | T |
| CV_1620 |  | conserved hypothetical protein | 20.96 | 0.71 | -4.88 | 1.80E-005 | T |
| CV_3260 |  | conserved hypothetical protein | 9.89 | 0.7 | -3.81 | 0 | T |
| CV_3505 |  | probable two-component response regulator | 25.7 | 0.79 | -5.02 | 0 | T |
| CV_2644 |  | probable two-component response regulator | 14.17 | 1.07 | -3.73 | 0 | T |
| CV_1947 | *czcR* | two-component response regulator | 11.76 | 0.79 | -3.9 | 0.01 | T |
| CV_3768 | *spoT* | guanosine-3',5'-bis(diphosphate) 3'-diphosphatase | 210.01 | 5.73 | -5.2 | 8.37E-013 | T |
| CV_0537 |  | hypothetical protein | 19.87 | 1.14 | -4.12 | 0.01 | T |
| CV_1220 |  | hypothetical protein | 43.03 | 4.7 | -3.19 | 0 | T |
| CV_0562 | *phoB* | phosphate regulon transcriptional regulatory protein phoB | 17.28 | 1.45 | -3.58 | 0 | T |
| CV_2323 |  | probable transcriptional regulatory protein, LuxR family | 63.35 | 5.66 | -3.48 | 8.76E-006 | T |
| CV_2399 |  | probable two-component system | 15.71 | 0.9 | -4.12 | 0.01 | T |
| CV_3341 |  | probable sensory transduction protein | 104.45 | 9.52 | -3.46 | 9.93E-009 | T |
| CV_3686 | *had* | histone deacetylase | 28.01 | 1.05 | -4.73 | 0 | T |
| CV_4091 | *cviI* | N-acyl homoserine synthase; autoinducer synthase, quorum sensing controlled system | 171.85 | 19.44 | -3.14 | 5.39E-005 | T |

^1^gene expression in cells grown for 24 h

^2^ gene expression in cells grown for 24 h in presence of 400 µg ml^-1^ of GLE

^3^ functional categories as per *C. violaceum* ATCC12472 database from the Brazilian Genome Virtual Institute of Genomic Research (BRGene) (http://www.brgene.lncc.br). [C, Energy production and conversion; D, Cell division and chromosome partitioning; E, Amino acid transport and metabolism; F,Nucleotide transport and metabolism; G, Carbohydrate transport and metabolism; H, Coenzyme metabolism; [I, Lipid metabolism](http://www.brgene.lncc.br/final/searchFinal.cgi?field=COGClassification&content=I&typeText=contain); [J, Translation, ribosomal structure and biogenesis](http://www.brgene.lncc.br/final/searchFinal.cgi?field=COGClassification&content=J&typeText=contain); [K, Transcription](http://www.brgene.lncc.br/final/searchFinal.cgi?field=COGClassification&content=K&typeText=contain); L, DNA replication, recombination and repair; M, Cell envelope biogenesis, outer membrane; N, Cell motility and secretion; O, Posttranslational modification, protein turnover, chaperones; P, Inorganic ion transport and metabolism; Q, Secondary metabolites biosynthesis, transport and catabolism; R, General function prediction only; S, Function unknown; T, Transduction mechanisms]
