# Supplementary material for: Guava Leaf Extract Inhibits Quorum-Sensing and Chromobacterium violaceum Induced Lysis of Human Hepatoma Cells: Whole Transcriptome Analysis Reveals Differential Gene Expression
Source: PLoS One. 2014 Sep 17;9(9):e107703. doi: 10.1371/journal.pone.0107703 (PMC4167859; doi:10.1371/journal.pone.0107703)
Supplement: File S1 — Method: Whole genome transcriptome analysis. (DOCX) [file pone.0107703.s003.docx]

**File S1**

**Whole genome transcriptome analysis**

**Isolation of total RNA devoid of rRNA**

1 ml of 24 hrs grown culture of control (cells grown without GLE) and experiment (cells grown with GLE) samples were harvested in 1.5 ml RNase free tube by centrifuging at 13000 rpm at 10 minutes at 4ºC and the pellet was used for RNA isolation using Ribopure bacteria kit (Ambion) as per manufacturer's protocol. Total RNA was checked on 1% agarose gel by loading 1μg and quantified on nanodrop 8000 spectrophotometer. Total RNA was used to enrich the RNA transcripts by selectively depleting ribosomal RNA molecules (rRNA) using MICROBExpress bacterial mRNA purification Kit as per protocol.

**Construction of cDNA Library followed by PCR amplification**

cDNA Library was prepared using the Ion Total RNA-Seq Kit v2 (Catalog Number 4475936). 500ng of rRNA depleted Total RNA (mRNA) was fragmented using RNase III enzyme followed by purification of fragmented RNA by AgenCourt beads. The fragmented rRNA depleted total RNA (mRNA) was hybridized with adapters and ligation was carried out. Reverse transcription was carried out to obtain cDNA from the RNA. Magnetic Bead Cleanup Module was used to purify cDNA followed by PCR amplification. Amplified cDNA was purified with Magnetic Bead Cleanup Module. The final amplified cDNA library was quantified and qualified using High Sensitivity DNA Chip on Bioanalyzer.

**Template preparation and sequencing run on Ion Torrent**

Template preparation and enrichment was performed as per Ion OneTouch™ 200 Template Kit (Cat no. 4471263) on automated Ion OneTouch platform as per protocol of Ion OneTouch system (publication part number 4472430 Rev. C). After enrichment the beads were loaded on Ion 318 Chip for 200 base-read sequencing using the Ion PGM 200 Sequencing Kit (Cat no 4474004) by following protocol of chip loading and run performing as publication number 4474246 Rev D to generate the sequence data. The raw reads generated were filtered using prinseq-lite program with QV > 20 and other contaminants such as adapters were trimmed. A total of 33,54,744 and 30,09,475 high quality reads for Control and Experiment sample respectively were used for transcriptome data analysis. The Ion Torrent data for Control and Experiment samples was mapped on the reference genome of *Chromobacterium violaceum* ATCC 12472 (http://www.ncbi.nlm.nih.gov/nuccore/AE016825.1) with genome size of 4.75 Mb.

**Differential gene expression in Control & Experiment samples**

There are 4,529 genes present in Chromobacterium violaceum ATCC 12472 GFF file. These genes were used to study the differential gene expression in Control & Experiment samples respectively by using the whole transcriptome analysis pipeline. The FPKM value for each gene was calculated for the combination of samples Control and Experiment. These FPKM values (FPKM_control and FPKM_experiment) were further used to calculate the log fold change as log_2_ (FPKM_experiment/FPKM_control). Moreover, uncorrected p-value of the test statistic for each gene was also computed. The analysis was carried out for commonly expressed genes reported in Control and Experiment samples respectively. These genes were further divided on the basis of their statistical significance (which can be either "yes" or "no", depending on whether p value is greater than the FDR after Benjamin-Hochberg correction for multiple-testing).
